# Supplementary material for: Optical-field driven charge-transfer modulations near composite nanostructures
Source: Nat Commun. 2020 Dec 1;11:6150. doi: 10.1038/s41467-020-19423-3 (PMC7708636; doi:10.1038/s41467-020-19423-3)
Supplement: Supplementary file 1 — Supplementary Information [file 41467_2020_19423_MOESM1_ESM.pdf]

Supplementary Information: Optical-Field Driven Charge-Transfer Modulations near Composite NanoStructures

Kwang Jin Lee, Elke Beyreuther, Sohail A. Jalil, Sang Jun Kim, Lukas M. Eng, Chunlei Guo, Pascal André

SI-I. Methods and Sample characterisations 1
SI-I.1. Preparation of the Samples 1
SI-I.1.a. Substrate Structures 1
SI-I.1.b. Donor:Acceptor Molecules and Thin Films 1
SI-I.2. Steady State Absorbance and Transient Absorption 2
SI-I.3. Transient Absorption Spectroscopy 2
SI-II. Time Resolved Surface Photovoltage Characterisation 2
SI-II.1. Setup and Experimental Protocol 2
SI-II.2. Further Characterisations 3
SI-II.3. Band Diagram Illustration 3
SI-III. Optical Field Intensity Determination 4
SI-III.1. Spectroscopic Ellipsometry 4
SI-III.2. Optical Constants 4
SI-III.3. Electric Field and Absorption Calculations 4
SI-IV. Further Transient Optical Characterisations 5
SI-IV.1. Charge Separation and Recombination Dynamic Comparison 5
SI-IV.2. Control Experiments 5
SI-IV.2.a. Transmittance vs Reflectance Modes 5
SI-IV.2.b. Donor:Acceptor and Metal Films in Direct Contact 5
SI-IV.2.c. Nanophotonic Structure vs Thick Metal Films 6
SI-V. Generalising Marcus Theoretical Framework with Nonlocal Enhanced Optical Field Effects 6
SI-V.1. Basics of Marcus Theory 6
SI-V.2. Image-Dipole Interactions 7
SI-V.3. Reorganisation Energy 7
SI-V.4. Optical Field Effects 7
SI-V.4.a. Optical Field Effect on the Gibbs Free Energy (ΔG) 8
SI-V.4.b. Optical Effect on the Reorganisation Energy (λ) 9
SI-V.4.c. Optical Effects on ΔG and λ 10
SI-V.5. Schematic Representations of Nonlocal Enhanced Optical Field Effects in Generalised Marcus Theory 11
SI-V.5.a. Potential Energy Surfaces 11
SI-V.5.b. Dependence of the Logarithm of the CT Rates 13
SI-V.6. Comments on the Generalisation Formalism 13
SI-VI. Control Experiments and NEOF Analysis Consistency Checks 13
SI-VI.1. Relative Contributions of the Pump and Probe Beams 13
SI-VI.2. Ruling out Non-Linear Optical Effects 14
SI-VI.3. NanoPhotonic and Thick Metal Films Structures 15
SI-VI.4. Tuning the Probe Beam Power and Diameter 16
SI-VI.5. Relative Contribution of the Number of Pairs on the Optical Field 16
SI-VI.6. Effect of the Donor:Acceptor Film Thickness 17
SI-VII. Comments and Outlook 17
SI-VIII. Glossary 17
SI-IX. Supplementary References 18

SI-I. Methods and Sample characterisations
SI-I.1. Preparation of the Samples
SI-I.1.a. Substrate Structures

The composite nanostructures (CNSs) were fabricated on fused silica by Korea Advanced Nano Fab Center (KANC) and at the University of Rochester. The deposition was completed by ion beam evaporation to form 10 nm thick metal and oxide successive layers with a maximum metal volume fraction $f_v = 0.5$ until the top-cover of Al_2O_3 was varied from 10 nm to 1 µm. Four pairs of bi-layers are the upper limit above which the transmitted probe beam used in the transient absorption experiments is too weak to be reliably measured. The validity of the measurements was confirmed in reflectance mode.

SI-I.1.b. Donor:Acceptor Molecules and Thin Films

Organic semiconductors were selected because of their relevance to electronics,[1-10] and the adjustability of the relative position of donor:acceptor (D:A) groups, in terms of energy levels and spatial positions. Indeed by adjusting molecular design and preparation conditions, covalently linked discotic D:A dyads present ordered supramolecular self-assemblies with nanometer size lamellar or columnar domains with segregated π-stacked molecules.[11-20] The resulting D:A interface is an attractive model system to investigate CS and CR mechanisms because of the reduced domain size and interface distributions, which otherwise can also impact on exciton formation, diffusion, dissociation and recombination and blur the variation of CT dynamics.

The dyad molecules were synthesised and purified as described in the literature.[18,19] SI-Figure 1 |a displays the chemical structure of the donor-acceptor molecule made of triphenylene and perylene diimide moieties chemically grafted with a flexible decyloxy flexible bridge to form the TriPh:PerDi dyad.

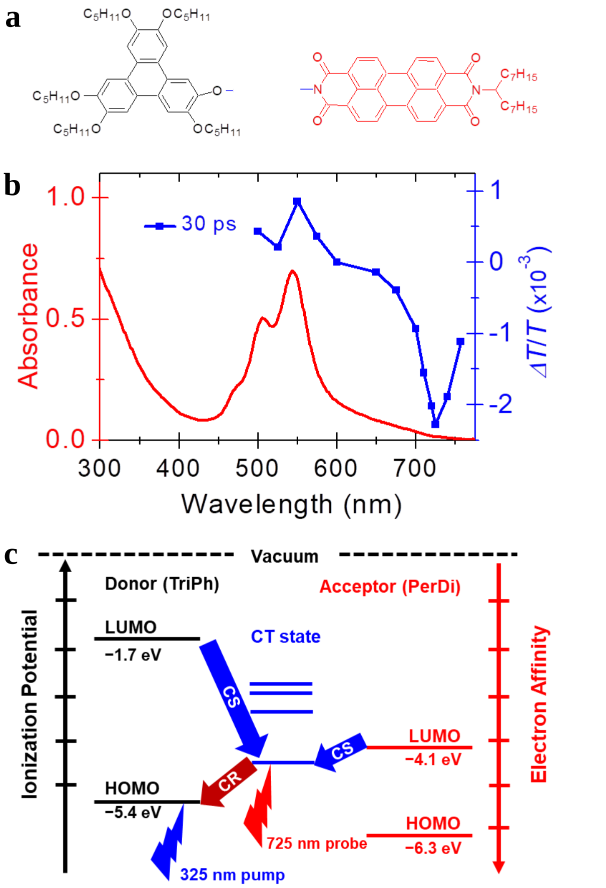

SI-Figure 1 | Characteristics of the D:A system. a, Molecular structure and energy level of the donor (Triphenylene) and acceptor (Perylene diimide) moieties. **b,** steady state absorbance (left, red) and transient absorption signal as a function of the probe wavelength (right, blue) measured with 325 nm pumping and 30 ps time delays. **c,** HOMO and LUMO energy levels of the D and A moieties, and illustration of the charge transfer process taking place in D:A film under photoexcitation.

The chloroform solution was spin-coated at 1 krpm directly on top of the CNSs. The self-assembly of the dyad molecules was optimised by an annealing treatment of 120 min at 120 °C on a hotplate and under ambient conditions. The thickness of the organic films was measured with a Bruker Dektak XT profilometer from NIST, leading to ~55 ± 5 nm value consistent with the 60 ± 6 nm thickness obtained with the spectroscopic ellipsometry measurements presented in section SI-III.1.

In such system, the discotic mesogenic conjugated units of the dyad self-assemble in a solid state multi-segregated D-A columnar structure. It is characterised by edge-on and parallel orientations compared to the surface of the substrate are observed for the molecules and the columns, respectively, a ~ 8 Å thick molten alkyl chain continuum insulating the conjugated core of the TriPh and PerDi columns from the Al_2O_3 interface, and an average distance of ~19.1 Å separates the columns.[18-20]

SI-I.2. Steady State Absorbance and Transient Absorption

UV-vis absorbance and reflectance spectra were measured using a Hitachi U-3310 spectrophotometer and the absorbance was determined with the standard A=-log$_{10}T$ equation and with fused silica substrates as a reference when needed.

SI-Figure 1 |b presents the normalised absorbance spectrum and transient absorption data of a D:A thin film spincoated on a fused silica substrate and briefly annealed in the fluid state of the mesophase to increase the regularity and planar orientation and reach long-range order. Broadened by π-π interactions, both TriPh and PerDi's characteristics appear at high and low energy, respectively. The tail above 600 nm is attributed to π-staking of the conjugated moieties, which leads to a very efficient photo luminescence quenching of the two moieties.

Transient absorption (TA) spectroscopy was used to monitor the CT state with a 325 nm pump. In SI-Figure 1 |b, the negative relative transmittance variation ($\Delta T/T$<0) observed at high energy corresponds to ground state bleaching, while the large positive signal ($\Delta T/T$>0) above 650 nm corresponds to the PerDi anion absorption,[18,20-23] which 725 nm maximum was used as the probe wavelength.

SI-Figure 1 |c illustrates the HOMO:LUMO energy levels of TriPh and PerDi moieties. It confirms that the former behaves as donors (-1.7:-5.4 eV), while the latter is the acceptor (-4.1:-6.3 eV), leading the formation of the $TriPh^+$:$PerDi^-$ charge transfer state. In this D:A films, the most favourable pathway is for a photoexcited electron to transfer from the TriPh's LUMO to the CT state, with no visible energy transfer from the D to the A units over the time scale covered by the transient absorption data.[18,20]

SI-I.3. Transient Absorption Spectroscopy

The femtosecond transient absorption measurements were carried out in transmittance mode, and identical dynamics were confirmed in reflectance mode. Both modes used pump and probe pulses generated by a Ti:Sapphire regenerative amplifier (Spitfire Pro XP, Spectra-Physics) working with an 800 nm output. A Mai Tai laser composed of a mode-locked Titanium-doped sapphire (Ti^{3+}:Al_2O_3) laser (Tsunami) and of a diode-pumped continuous wave Nd:YVO_4 laser (Millennia). The former was used as the seeding laser for the regenerative amplifier. The latter was used to pump the Tsunami. The regenerative amplifier was based on a Q-switched intra-cavity frequency doubled Nd:YLF laser operating at a repetition rate of 5 kHz and delivered 60 fs long pulse centered at 800 nm. The 325 nm pumping beam was obtained by 4th harmonic generation using two beta barium borate (BBO) crystals after the optical parametric amplifier (TOPAS-Prime, Spectra-Physics) operating at 1300 nm. The pump beam was attenuated to 2.0 mW using neutral density filters in front of the sample. The 725 nm probe beam was selected from a white light continuum probe generated in the visible range with half of the 800 nm regenerative amplifier output power and a 2 mm thickness sapphire window. The time delay between the pump and probe beam was varied up to 900 ps by using a delay line. The time intervals used for the charge separation and recombination measurements were ~100 fs and ~10 ps in stepping motor, respectively. The pump beam was modulated using a mechanical chopper working at 220 Hz and the differential transmittance $\Delta T/T$ of the probe beam was determined as a function of the delay time with a photodiode and a lock-in detection. A filter (FSR-RG645, Newport) cutting the light below 640 nm was used to reduce the potential impact of scattered light from the pump beam. The pump (λ = 325 nm, P ~ 2 mW, D ~ 1 mm) and probe (λ = 725 nm, P ~ 20 μW, D ~ 200 μm ; unless stated otherwise) beams hit the substrates on the D:A-CNS side and he differential transmittance was calculated as

$$\frac{\Delta T}{T}(\lambda,t)=\frac{T_{on}(\lambda,t)-T_{off}(\lambda,t)}{T_{off}(\lambda,t)} \quad \text{(SI-1)}$$

where T_{on} and T_{off} correspond to the sample transmittance with the pump beam on and off, respectively. The charge separation and recombination characteristic times were deduced from the $\Delta T/T$ curves plotted as a function of the delay time and fitted with eqs. SI-2 and SI-3 for CS and CR, respectively.

$$\frac{\Delta T}{T}(t)=\alpha.\left[1-exp\left(-\frac{t}{\tau_{CS}}\right)\right] \quad \text{(SI-2)}$$

$$\frac{\Delta T}{T}(t)=-\alpha.exp\left(-\frac{t}{\tau_{CR}}\right) \quad \text{(SI-3)}$$

All the D:A film transient spectra displayed a single exponential behaviour. The characteristic times obtained in transmittance and reflectance modes are identical within 5 %, which corresponds to the experimental uncertainty. The fits were completed using non-linear least squares procedures included in Kaleidagraph and Origin.

SI-II. Time Resolved Surface Photovoltage Characterisation

SI-II.1. Setup and Experimental Protocol

All surface photovoltage measurements were carried out with a Kelvin probe (KP) setup, as described in detail in ref. [24]. Three different samples were investigated, all of them consisting of a fused silica substrate and 4 pairs of Ag/Al_2O_3 bilayers, while their Al_2O_3 top layer was varied in thickness (10 nm, 125 nm, 300 nm). The samples were cleaned in ultrasound bath (1 min acetone, followed by isopropyl alcohol), dried in a nitrogen gas stream, and back-contacted/glued with conductive silver paste to the sample holder of the KP. The contact potential difference (CPD) between a semi-transparent gold electrode and the sample surface was recorded in the dark and under 633 nm illumination from a He-Ne laser (JDS Uniphase, max. output power: 10 mW). The power reaching the sample surface was 3 mW with a spot size diameter of 2 mm. For the two thicker Al_2O_3 layers, the CPD was monitored for 5 min in the dark to derive a CPD average dark value, followed by 30 min under illumination, and again 30 min in the dark, with data points recorded every second. The corresponding "light-ON" and "light-OFF" CPD transients were acquired at least three times and averaged to eliminate environmentally-induced fluctuations. Subsequently, a further 4-point average was performed, as illustrated in SI-Figure 2 showing both the data before and after averaging. The 10 nm Al_2O_3 top layer sample showed a much faster SPV response. Then, the illumination and darkness steps were only 10 min, since SPV saturation was achieved within this time interval. All SPV diagrams depict the SPV absolute value, also referred to as "SPV amplitude" in the main text, that was calculated based on the following equation:

$$SPV=\left|CPD_{dark}-CPD_{bright}\right| \quad \text{(SI-4)}$$

Whilst beyond the scope of the present work, the determination of the exact sign of the SPV would need a detailed knowledge on the band alignment across the CNSs, including realistic values for the

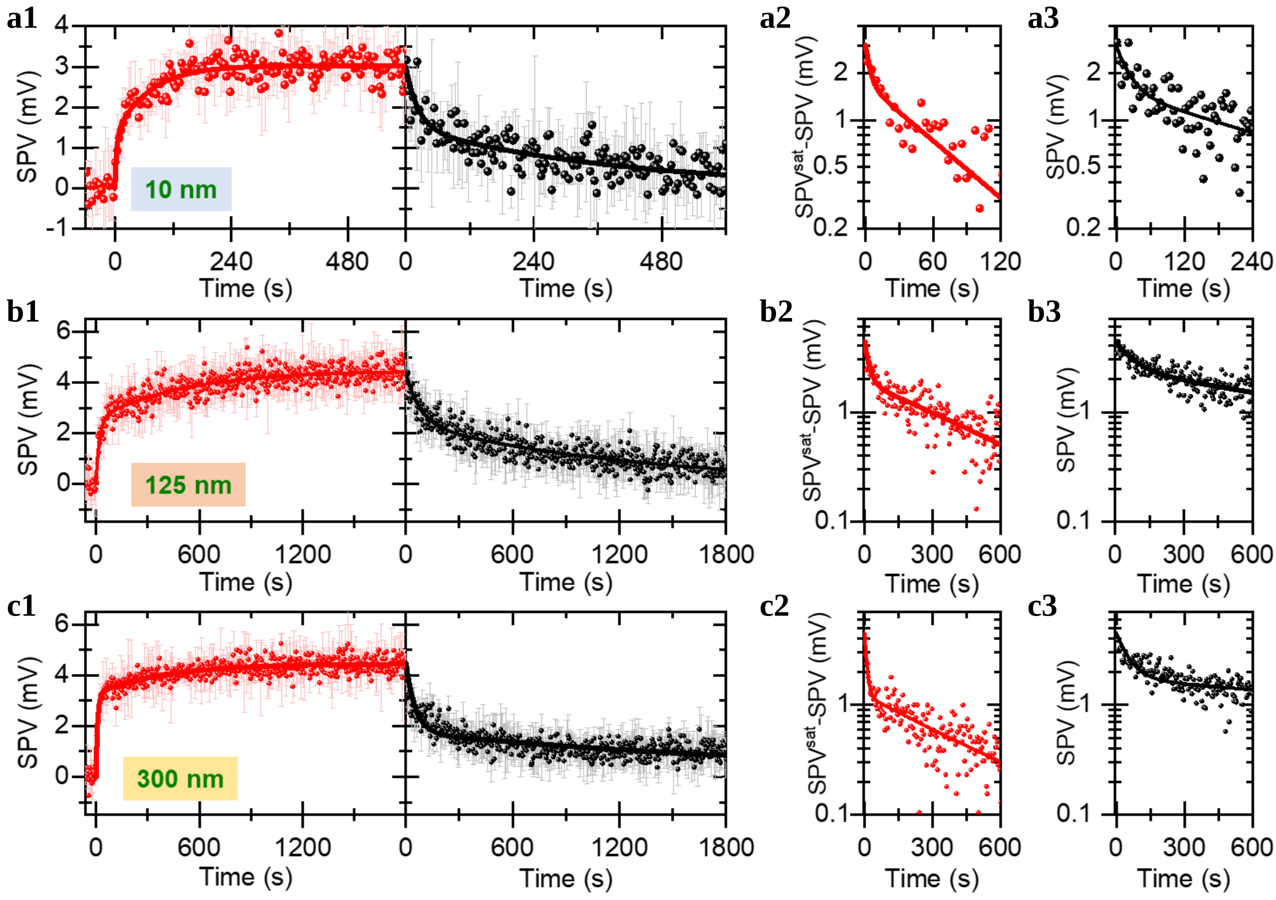

SI-Figure 2 | Surface Photovoltages Dynamics and Amplitudes across CNSs. Top Al_2O_3 film thickness of **a,** 10 nm **b,** 125 nm, and **c,** 300 nm. **1,** Typical increase and decrease of the SPV signal as a function of time upon ON (red) and OFF (black) switching of a laser beam. Semi-log plots of **2,** the SPV rise, and **3,** SPV signal recovery. Note that no signal could be measured on single Ag thin film grown on a FS substrate, showing that interface states between the FS substrate and the first Ag layer are not relevant for the SPV built-up. All the measurements were completed on bare 4p Ag-Al_2O_3 CNSs, with a 633 nm photo-excitation and at ambient but clean-room conditions, with stabilized temperature and air humidity.

electron affinity and work function of the aluminium oxide layers. This would allow deriving the Fermi level position and thus reconstructing the band alignment at all the interfaces to the Ag metal layers.

SI-II.2. Further Characterisations

Wide-bandgap oxides can show very complicated SPV transients due to multiple and entangled charge transitions,[25] which in the most complicated case can be only numerically approximated or partially exponentially fitted. In the CNSs explored in this work, the SPV-vs-time data observed could be fitted by bi-exponential equations, as illustrated in SI-Figure 2. This takes into account that there are obviously at least one fast and one slow component involved in the SPV mechanism.

For the light-ON SPV-transients, the following relation was used:

$$SPV_B = SPV_{sat}\left(1 - e^{-\frac{t}{t_{SB}}}\right) - A_B\left(e^{-\frac{t}{t_{SB}}} - e^{-\frac{t}{t_{FB}}}\right) \qquad \text{(SI-5)}$$

with “B” standing for “bright”, SPV_{sat} being the SPV absolute value at saturation, A_B is a coefficient between zero and SPV_{sat}, and t_{FB} and t_{SB} are the characteristic times for the fast and the slow component, respectively.

The light-OFF transients were analogously approximated by:

$$SPV_D = SPV_{sat}\, e^{-\frac{t}{t_{SD}}} + A_D\left(e^{-\frac{t}{t_{FD}}} - e^{-\frac{t}{t_{SD}}}\right) \qquad \text{(SI-6)}$$

where “D” stands for “dark”.

We note that, due to the very large bandgap of aluminium oxide of about 7 eV and the much smaller excitation energy of 1.96 eV corresponding to the 633 nm laser wavelength, any “intrinsic” part of the SPV effect must stem from charge transfer processes between the bands and one or more in-gap states. While extrinsic contributions to the SPV signal due to physi-/chemisorbed species at the surface cannot be completely ruled out, these are assumed to be weak, since they would not allow for any strong variation of the SPV amplitude and the time constants with the aluminium oxide thickness in contrast of what is observed in this work. An in-depth study of the gap-state distribution and their capture cross sections for photons and electrons/holes would require a separate study, following an similar approach as in ref. [25].

SI-II.3. Band Diagram Illustration

SI-Figure 3a shows a possible configuration of the Al_2O_3-air interface with a downward band bending towards the surface corresponding to a hole-depleted interface in the p-type-like oxide.[26] Under illumination, the incident photons excite electrons from the valence band to the trap levels, the corresponding holes move towards the bulk due to the internal field, and the charge redistribution between surface and bulk alters the band bending and the surface potential. The difference of the total surface band bendings in the dark and under illumination corresponds to the surface photovoltage (SPV).

SI-Figure 3b shows a possible configuration of the composite nanostructure-air interface with the Al_2O_3 configuration influenced by the Ag layers underneath, which can also build up a depletion zone due to ionic bonding from the silver layer to outer oxygen ions, which means an electron transfer from Ag to O, the latter ions act thus as acceptors.[27-29] In this case, all optically accessible buried Ag/Al_2O_3 interfaces can also show an SPV contribution (for

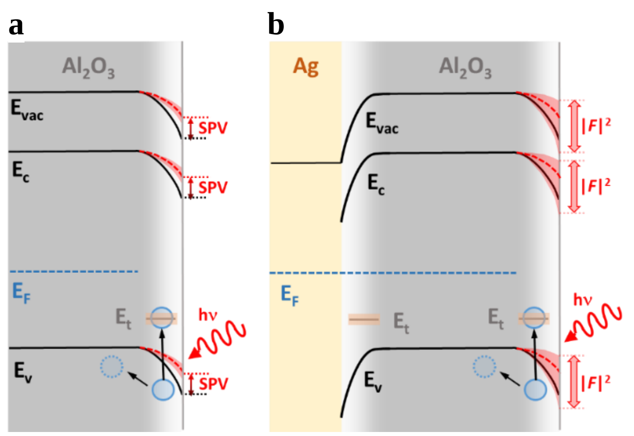

SI-Figure 3 | Schematic illustration of a tentative band diagram of the present system. a, aluminium oxide, **b,** composite nanostructure. With E_{vac} the vacuum level, E_c the conduction level, E_F the Fermi level, E_t a trap level distribution, E_v the valence level. The red dashed curve illustrates an SPV effect on band bending, the light red cone illustrates the SPV range under the influence of the optical field effect. The areas in grey, grey to white gradient and yellow stand for the oxide, hole-depletion region, and metal respectively.

the sake of clarity, the SPV process is only sketched for the surface); the measured SPV is a cumulative effect, since all interfaces of the structure are connected in series.

SI-III. Optical Field Intensity Determination

SI-III.1. Spectroscopic Ellipsometry

A spectroscopic ellipsometer from Ellipso technology Co. Inc. was used in the measurement of the rotating polariser type. The spectroscopic ellipsometry (SE) measurements were performed in the spectral range of 1.2-5.2 eV (~ 240-1000 nm) for 60°, 65° and 70° incidence angles. This approach improves the accuracy of the calculations allowing the determination of film thicknesses and optical refractive index values. The measured ellipsometric angles Ψ and Δ are defined from the ratio of the reflection coefficients r_p and r_s for the p- and s- polarisations, respectively, (i.e., polarisation of the electric field parallel and perpendicular to the plane of incidence) according to

$$\frac{r_p}{r_s} = tan(\psi).e^{i\Delta} \quad \text{(SI-7)}$$

From the analysis of the SE measurement data, the dielectric function of a specific material is determined. For this, the optical response of the measured samples was modelled in the Tauc-Lorentz dispersion formula,[30] which included multiple oscillators.

$$\varepsilon_{i,TL}(E) = \varepsilon_{i,L} x \varepsilon_{i,T} \quad \text{(SI-8)}$$

$$\varepsilon_{i,TL}(E) = \frac{1}{E}\frac{AE_oC(E-E_g)^2}{(E-E_o)^2+C^2E^2} \quad \text{for } E > E_g$$

$$\varepsilon_{i,TL}(E) = 0 \quad \text{for } E \leq E_g$$

$$\varepsilon_r(E) = \varepsilon_r(\infty) + \frac{2}{\pi}p\int_{E_g}^{\infty}\frac{\xi.\varepsilon_i(\xi)}{\xi^2 - E^2}d\xi \quad \text{(SI-9)}$$

The experimental ellipsometry data were fitted using the Levenberg-Marquardt algorithm for minimizing the mean-squared error (*MSE*).

$$MSE = \frac{1}{2N-M}\sum_{i=1}^{N}\sqrt{\left(\alpha_i^{mod}-\alpha_i^{exp}\right)^2 + \left(\beta_i^{mod}-\beta_i^{exp}\right)^2} \quad \text{(SI-10)}$$

where N is the number of (α, β) pairs, M is the number of fitted parameters in the model. The superscripts *mod* and *exp* indicate model-generated and experimental data, respectively.

SI-III.2. Optical Constants

SI-Figure 4 | presents the permittivity of each compound as deduced from ellipsometry measurements. There used to calculate the properties of the samples, including substrates and D:A thin films. We note that whilst optical analyses presented in the following part of the SI remain partially subject to the models and fits used to analyse the spectroscopic ellipsometry data, they do highlight the potentials of considering carefully the effects of optical fields when D:A systems are located near CNSs.

SI-III.3. Electric Field and Absorption Calculations

The invariant imbedding method is a powerful tool for handling

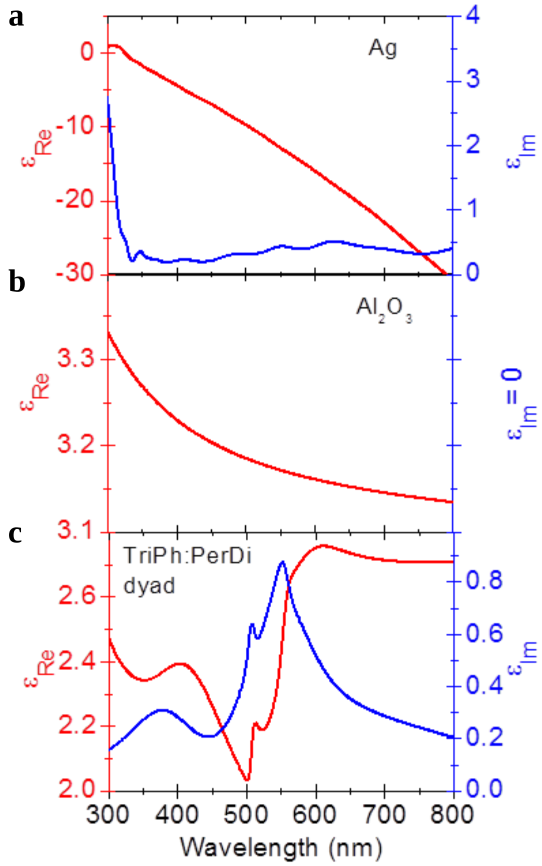

SI-Figure 4 | Permittivity used to calculate the optical field. a, Silver, **b,** aluminium oxide, and **c,** TriPh:PerDi dyad thin film. Left and right sides present the real (red) and imaginary (blue) components spectral variation of the permittivity.

the propagation of electromagnetic waves in one-dimensional inhomogeneous media. The Maxwell's equations were applied to the amplitude of the electric field. The exact differential equations satisfied by the reflection coefficient and the electric field amplitude were obtained with respect to medium size. These were supplemented with the initial conditions from Fresnel formulas.[31-33] Using this method, the exact solutions for the reflection and transmission coefficients of incident waves and electric field distribution inside the organic photovoltaic (OPV) media are obtained.[8]

Let's assume that the wave is incident from the region where $z > L$ and is transmitted to the region where $z < 0$. The dielectric permittivity is assumed to be given by

$$\varepsilon(z) = \begin{cases} \varepsilon_1, for\ z > L \\ \varepsilon(z), for\ 0 \leq z \leq L \\ \varepsilon_2,\ for\ z < L \end{cases} \quad \text{(SI-11)}$$

We consider a plane wave of unit magnitude

$$\tilde{E}(x,z) = E(z)exp(iqx) = exp[ip(L-z) + iqx] \quad \text{(SI-12)}$$

with

$$p = \sqrt{\varepsilon_1}k_0 cos\theta \quad \text{(SI-13a)}$$
$$q = \sqrt{\varepsilon_1}k_0 sin\theta \quad \text{(SI-13b)}$$

where θ is an incident angle.

The reflection and transmission coefficients are defined by the wave function outside the medium:

$$\tilde{E}(x,z) = \begin{cases} [e^{ip(L-z)} + r(L)e^{ip(z-L)}]e^{iqx}, z > L \\ t(L)e^{-ipz+izx}, \quad z < 0 \end{cases} \quad \text{(SI-14)}$$

Using the invariant imbedding method, the exact differential equations of r and t can be obtained:

$$\frac{1}{i\sqrt{\varepsilon_1}k_0}\frac{dr}{dl} = 2r(l)cos\theta + \frac{\tilde{\varepsilon}(l)-1}{2cos\theta}[1+r(l)]^2 \quad \text{(SI-15)}$$

$$\frac{1}{i\sqrt{\varepsilon_1}k_0}\frac{dt}{dl} = t(l)cos\theta + \frac{\tilde{\varepsilon}(l)-1}{2cos\theta}[1+r(l)t(l)]^2 \quad \text{(SI-16)}$$

where

$$\tilde{\varepsilon} = \varepsilon/\varepsilon_1 \quad \text{(SI-17)}$$

Based on these equations, we can obtain the exact differential equation of electromagnetic field distribution as follow:

$$\frac{1}{i\sqrt{\varepsilon_1}k_0}\frac{dE(z;l)}{dl} = 2cos\theta E(z;l) + \frac{\tilde{\varepsilon}(l)-1}{2cos\theta}[1+r(l)]E(z;l) \quad \text{(SI-18)}$$

For a given ($0 < z < L$), the field amplitude E(z ; L) is obtained by integrating this equation from $l = z$ to $l = L$ using the initial condition E(z ; z)=1 + r(z). In this study, we take normal incidence (θ =0) into account.

It is also noticeable that the quasi-static field approximation is only valid if an electromagnetic wave propagates over a characteristic length (L) of the system in a time (L/c) that is short compared to times of interest (τ). This leads to the condition $L/c << \tau$, where c is the speed of light in the propagation media. For a 725 nm light beam in a 1 µm thick Al_2O_3 spacer (n = 1.75 as determined in the present work by spectroscopic ellipsometry), $n.L/c$ is equal to ~ 5.8 fs, which is small compared to the probe pulse duration of ~ 60 fs. This shows that the CNSs can be properly described within the quasi-static field approximation. The quasi static assumption is widely used in the literature of nano/micro structures and CT.[34-37] Light trapping is consistent and supports our model being developed on the quasi-static field approximation.

SI-IV. Further Transient Optical Characterisations

SI-IV.1. Charge Separation and Recombination Dynamic Comparison

SI-Figure 5 | illustrates that CS and CR are anti-correlated with CR increasing while CS decreases, and vice-versa, when the top-thickness of the dielectric cover of the CNSs is tuned.

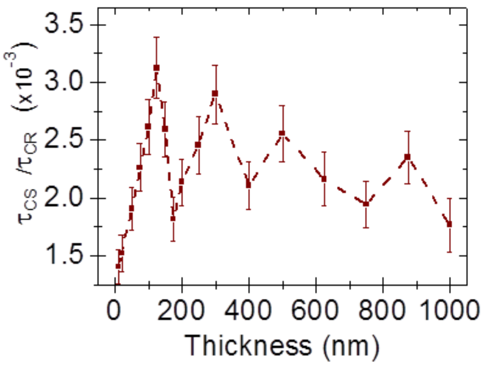

SI-Figure 5 | Quantitative TA analysis. Separation to recombination characteristic time ratio in D:A annealed thin films as a function of the thickness of the Al_2O_3 top-cover in 4p CNS.

SI-IV.2. Control Experiments

SI-IV.2.a. Transmittance vs Reflectance Modes

SI-Figure 6 | shows that the CR dynamic is independent of the measurement mode. Transmittance and reflectance mode measurements lead to 269 ± 5 and 270 ± 5 ps, respectively, with all the experimental parameters (excitation and probe wavelengths, power and beam diameters) kept constant.

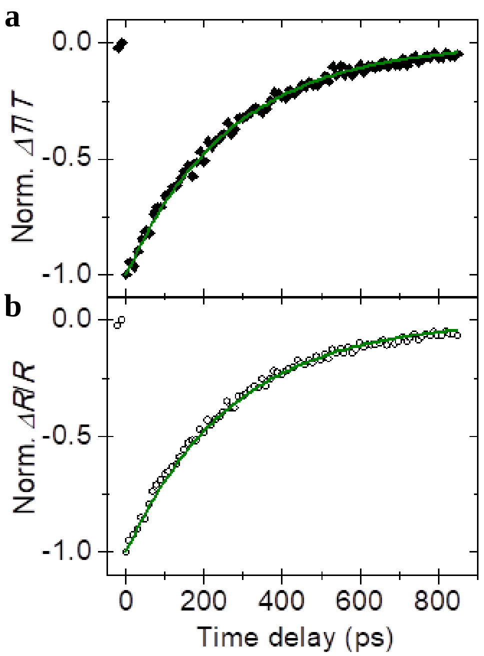

SI-Figure 6 | Transient spectroscopy modes. a, transmittance and **b,** reflectance mode measurements of charge recombination dynamics in annealed dyad thin films on a 125 nm Al_2O_3 top-cover CNS. Symbols: experimental data points, full lines: single exponential fits.

SI-IV.2.b. Donor:Acceptor and Metal Films in Direct Contact

As shown in SI-Figure 7 | and SI-Table 1, the CS and CR dynamics are unaffected by the presence or the removal of 10 nm thick Al_2O_3 cover on top of the metal layer. This is consistent with both the relatively large thickness of the Al_2O_3 top-cover and the alkyl-chain melt surrounding the dyad columns. We note that the mechanism of charge transfer to the substrate would have explained neither the non-monotonous CTD variations with the thickness of the dielectric cover, nor the CS and CR anti-correlation.

SI-Table 1. Charge separation (τ_{CS}) and recombination (τ_{CR}) times on selected substrates.

substrate	τ_{CS} (ps) *	τ_{CR} (ps) **	τ_{CS}/τ_{CR} (x10^{-3})
**FS-D:A ^{T}**	0.23 ± 0.05	226 ± 5	1.0 ± 0.2
**200 nm Ag ^{R}**	0.29 ± 0.05	276 ± 5	1.1 ± 0.2
**1 p_{10}-D:A ^{T}**	0.30 ± 0.05	256 ± 5	1.2 ± 0.2

* 100 fs and ** 10 ps time interval. ^{T} transmittance and ^{R} reflectance modes.

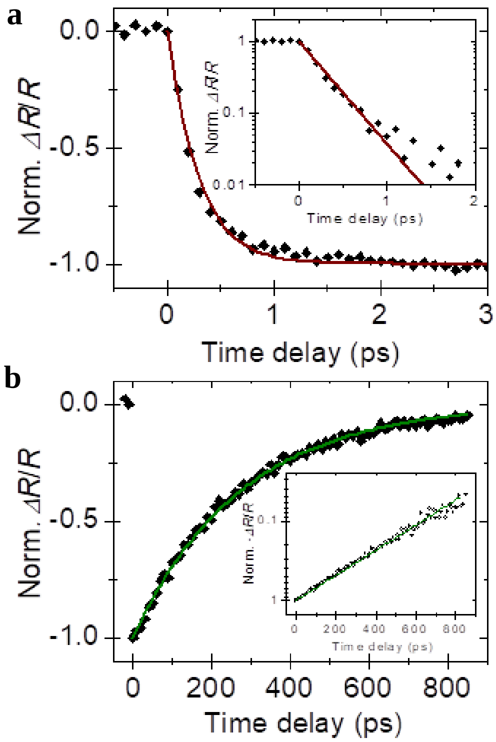

SI-Figure 7 | Transient absorption measurements of D:A molecules in direct contact with a silver metal film. a, Charge separation and **b,** recombination dynamics of annealed D:A thin films on 200 nm thick Ag substrates without Al_2O_3 top-cover. Relative reflectance variation as a function of the time delay (inset: semi-log scale of 1-$\Delta R/R$, and -$\Delta R/R$, note that to illustrate the signal variation the scales in the inset are inverted). Measurements were completed with 100 fs and 10 ps time delay steps for CS and CR, respectively. Symbols: experimental data points ; Solid lines: single exponential fits.

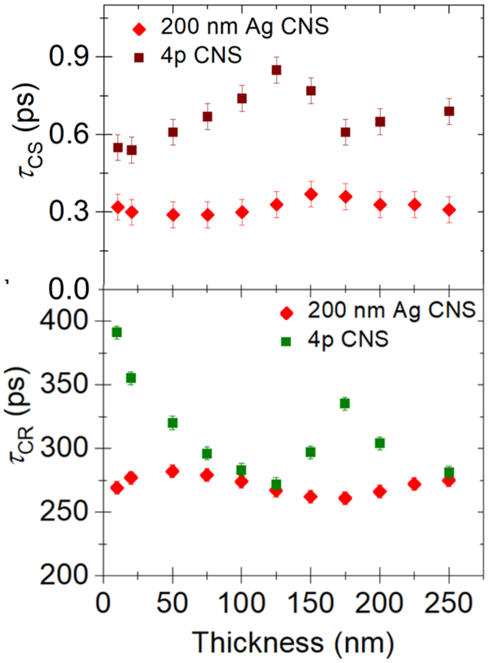

SI-Figure 8 | Charge transfer dynamics on 4p nanophotonic and plain metal substrates. a, Charge separation and **b,** recombination dynamics in annealed D:A thin films on 200 nm thick metal substrates (♦) and CNSs (■) covered with Al_2O_3 top-covers.

SI-IV.2.c. Nanophotonic Structure vs Thick Metal Films

SI-Figure 8 | shows weak charge transfer dynamic modulations on 200 nm thick Ag film when compared with 4p CNSs covered by the same Al_2O_3 top-covers. CS and CR modulations are smaller and only slightly larger than the measurement and fit uncertainty, respectively. Furthermore, it is noticeable that the periodicity of the CT modulations differs on 200 nm thick Ag film and 4p CNS, as the maxima and minima are obtained for different Al_2O_3 thicknesses.

SI-V. Generalising Marcus Theoretical Framework with Non-local Enhanced Optical Field Effects

SI-V.1. Basics of Marcus Theory

In a dielectric continuum, non-adiabatic CT reaction rates, k_{CT}, can be described in Marcus theory framework in terms of Planck constant (h), total reorganisation energy (λ_{CT}), thermal energy (k_BT), electronic coupling between the initial and final states (V_{DA}, CT integral), activation Gibbs free energy (ΔG^*_{CT}), and the Gibbs free energy gain (ΔG_{CT}, driving force) as stated in eq. SI-19 and SI-20:[18,38-40]

$$k_{CT} = \left(\frac{4\pi^3}{h^2\lambda_{CT}k_BT}\right)^{1/2} |V_{DA}|^2 exp\left(-\frac{\Delta G^*_{CT}}{k_BT}\right) \quad \text{(SI-19)}$$

$$\Delta G^*_{CT} = \frac{(\lambda_{CT}+\Delta G_{CT})^2}{4\lambda_{CT}} \quad \text{(SI-20)}$$

For illustration purposes, these are represented in SI-Figure 9 |, with the parabolic energy levels of the reactant and the product shifted along the reaction coordinates.

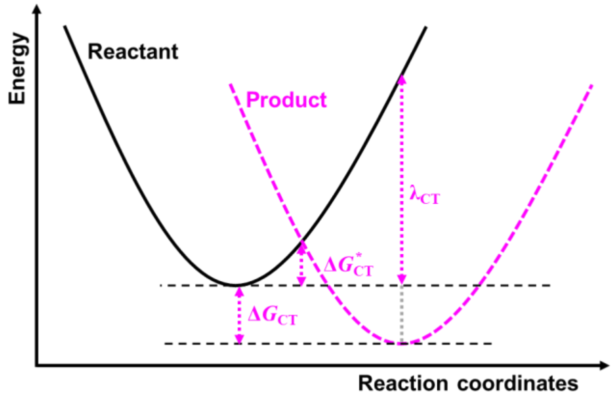

SI-Figure 9 | Typical energy diagram of a charge transfer. ΔG_{CT}, . ΔG^*_{CT}, and λ_{CT} are the driving force, activation energy, and reorganization energy, respectively.

The driving force, ΔG, is basically quantified by the offset between the minimum energy of reactant and that of the product. In the present case, these are the Donor's excited state (D^*:A) and the charge transfer state (D^+:A^-), respectively, while ΔG is given by the offset between the lowest unoccupied molecular orbital (LUMO) of the Donor and the LUMO of the acceptor. ΔG^*_{CT} is the activation Gibbs free energy, i.e. the energy barrier to be overcome for the reaction to occur. λ is the reorganization energy, which is the amount of energy needed to reorganize the nuclear configuration when a charge transfer takes place. This is indicated as the energy difference between the product's minimum energy and the reactant energy at nuclear position of the product ground state.

From Eq. (20), the parabolic relation predicts a maximum of k_{CT} at the barrier less point, i.e. where $-\Delta G_{CT} = \lambda_{CT}$. In the 'normal' region, for which $-\Delta G_{CT} < \lambda_{CT}$, any driving force increase (i.e. more negative ΔG_{CT}) reduces ΔG^*_{CT}, hence increases k_{CT}. In the 'inverted' region, defined as $-\Delta G_{CT} > \lambda_{CT}$, any driving force increase reduces k_{CT} as the activation energy decreases.

SI-V.2. Image-Dipole Interactions

In the Marcus theory framework extended to CNSs, CT state dipoles and their image potentials in the CNSs are included as a perturbation energy term:[18]

$$\delta G_{CT}^{IDI} = \Delta G_{CT}^{IDI} - \Delta G_{CT}^{Al_2O_3} \quad \text{(SI-21)}$$

The logarithm of the CNS to Al_2O_3 CT reaction rate ratio is characterised by δG_{CT}^{IDI} linear and quadratic terms:

$$ln\frac{k_{CT}^{IDI}}{k_{CT}^{Al_2O_3}} \cong -\alpha_{CT}\left[2\left(\lambda_{CT} + \Delta G_{CT}^{Al_2O_3}\right)\delta G_{CT}^{IDI} + \delta G_{CT}^{IDI\,2}\right] \quad \text{(SI-22)}$$

with $\alpha_{CT} = (4\lambda_{CT}k_BT)^{-1}$. Noticeably, eq. SI-22 applies to both CS and CR once the relation between the energy differences is accounted for. The perturbation induced by the CNS is:

$$\delta G_{CS-\gamma}^{IDI} = -\delta G_{CR-\gamma}^{IDI} = -\Delta\Phi_{\gamma}^{IDI}\frac{e^2}{4\pi\varepsilon_o}\Delta_{1/R} \quad \text{(SI-23)}$$

where γ is the dipole orientation parameter, $\Delta_{1/R}$ has the dimension of the inverse of a distance, is expected to be relatively independent of the substrates and is defined by analogy with solution related equations as:

$$\Delta_{1/R} = \frac{1}{R_{CC}} - \frac{1}{2r_{D+}} - \frac{1}{2r_{A-}} = \frac{1}{R_{CC}} - \frac{1}{r_{\pm}} \quad \text{(SI-24)}$$

$\Delta\Phi_{\gamma}^{IDI}$ is defined as

$$\Delta\Phi_{\gamma}^{IDI} = \frac{1}{L-\lambda}\int_{\lambda}^{L}\left(\phi_{\gamma}^{IDI} - \phi_{\gamma}^{Al_2O_3}\right)dr \quad \text{(SI-25a)}$$

$$\Delta\Phi_{\gamma}^{HMM} = \frac{1}{\varepsilon_{\gamma}^{IDI}} - \frac{1}{\varepsilon_{\gamma}^{Al_2O_3}(=1)} \quad \text{(SI-25b)}$$

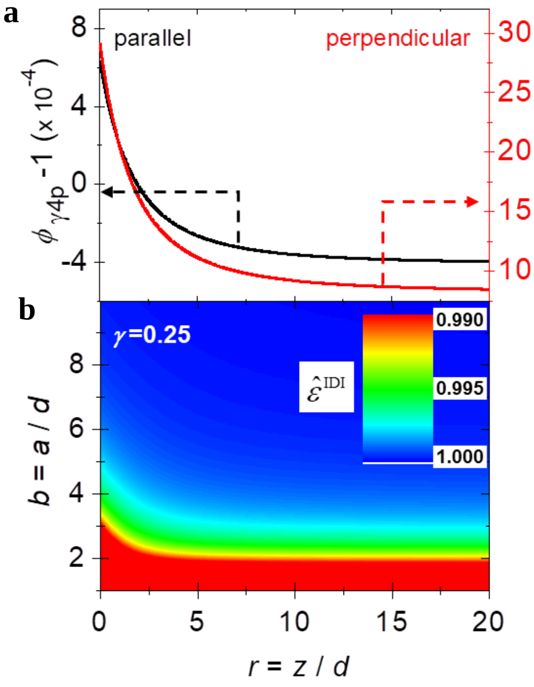

SI-Figure 10 | Image dipole interactions as a function of the normalised dipole-CNS interface distance. a, Dimensionless energy parameter (ϕ) for normalised dipole-CNS interface distance $b = a / d \approx 5$ and for dipoles parallel (black) and perpendicular (red) orientations to the substrate. a is the CNS layer thickness and d is the CT dipole length. **b,** False-colour plot of the nonlocal image dipole interactions manipulated permittivity as a function of the normalised dipole-CNS interface distance b for a dipole-substrate orientation $\gamma = 0.25$. Calculations were completed in 4p CNSs with a 10 nm thick Al_2O_3 top layer.

with ϕ, the dimensionless energy parameter corresponding to the ratio of the sum of the dipole energy and the image dipole interaction energy by the dipole energy, and which is the inverse of the CNS image dipole interactions manipulated permittivity, ε^{IDI}, so that CNS effects are always a function these parameters. In terms of formalism, ε^{IDI} corresponds to the average over the thickness of the D:A blend films, while $\hat{\varepsilon}^{IDI}$ is defined at a specific distance from the CNS. This approach describes qualitatively well the nonlocal effect associated with the presence of nearby CNSs.[18] We note that ε^{IDI} could also be labelled as nonlocal permittivity.

SI-Figure 10 | presents calculations completed for a 4 pair CNS with a 10 nm top layer of Al_2O_3. SI-Figure 10 |a shows that, whilst a stronger variation is associated with dipoles perpendicular to the CNS interface, regardless of the dipole orientation, image dipole interactions do not lead to any oscillatory behaviour which could otherwise have been found in the charge transfer dynamics as described in eq. SI-22. SI-Figure 10 |b confirms this for a range of dipole normalised CNSs as $\hat{\varepsilon}^{IDI}$ varies monotonously as a function of the CNS interface distance.

SI-V.3. Reorganisation Energy

The reorganisation energy includes two contributions: λ_{intra}, an intramolecular term accounting for vibrational, bond length and angles change within the reagents, i.e. donor and acceptor moities, and λ_{env}, accounting for reorientation and change of polarisation around the reagents upon CT. It can be expressed as

$$\lambda_{CT} = \lambda_{intra} + \lambda_{env} \quad \text{(SI-26)}$$

$$\lambda_{intra} = \frac{1}{2}\sum_{j} k_j \Delta q_j^2 \quad \text{(SI-27)}$$

Typical values for large aromatic molecules like perylene diimide range from λ_{intra} ~ 0.25 to 0.30 eV.[41-44] Calculations for triphenylene cationic derivatives suggest λ_{intra} ~ 0.18 to 0.32 eV.[45] In solution, assuming point charges, dielectric continuum description of the solvent, and an isotropic system, λ_{env} can be determined by the Born–Hush equation often used to describe solution CT.[38,39]

$$\lambda_{env} = \frac{e^2}{4\pi\varepsilon_o}\left(\frac{1}{2r_{D+}} + \frac{1}{2r_{A-}} - \frac{1}{R_{CC}}\right)\left(\frac{1}{\varepsilon_{optics}} - \frac{1}{\varepsilon_{static}}\right) \quad \text{(SI-28)}$$

with R_{cc}, the center-to-center distance between the donor and the acceptor, $r_{D+/A-}$ the ionic radii of the donor and the acceptor respectively, $\varepsilon_{optics}=n^2$ and ε_{static} the optical and static dielectric constants of the surrounding media, respectively. Whilst beyond the scope of this work, it can be noticed that the point dipole, homogeneous and isotropic media, center-to-center distance between the donor and the acceptor, spherical ions of $r_{D+/A-}$ radii related hypotheses implied in eq. SI-27 are not necessarily valid in thin films for which the expression of λ_{env} is not as simple as stated above.[45-47] Let us note that taking typical values for R_{cc} ~ 1.6 ± 0.1 nm, r_{TriPh^+} ~ 0.55 nm and r_{PerDi^-} ~ 0.76.

For films, the situation is then more difficult than in solution, and DFT type of calculations would be needed. Crystallinity and neighbours need to be taken into account as their presence decreases the reorganisation energy due to both molecular and electronic polarisation.[45,48,49]

SI-V.4. Optical Field Effects

A chromophore is very sensitive to its close environment so that both its photoluminescence amplitude and lifetime can be altered by viscosity, pressure, temperature, confinements and chemical activation.[50-57] In addition, we commented briefly in the main text that optical modulations have been reported for radiative (photoluminescent) dipoles as probed through fluorescence lifetime, quantum efficiency and energy transfer modulations near metal interfaces.[33,58-71] However we stressed that transition (radiative) dipoles

and non-radiative (quasi-static) dipoles are of different nature, and are then described by very different formalisms and theories.

Furthermore, it should be noticed that whilst the literature does refer to charge transfers involving non-radiative (quasi-static) dipoles, its focus has mostly been related to

i) CT to/from a metal surface, and

ii) image–charge interactions causing the occupied and unoccupied state energy to shift.[72]

To the best of our knowledge, there are no reports referring to photoinduced anti-correlated CTD modulations.

Supporting further the analysis presented below, we note that external static electric potentials are used to tune semiconductor properties such as photo-luminescence,[73-93] and electronic devices.[94-103] The effect of these external static electric field are usually proportional to F, F^2 or a combination of both depending upon the system under consideration. Leaving aside the differences between static and optical electric field, we explored how the formalism of the latter can be applied to the former to explain our original experimental results, and how this formalism can be integrated into a generalised theoretical framework.

SI-V.4.a. Optical Field Effect on the Gibbs Free Energy (*ΔG*)

CT states are quasi-static dipole moments, which see their free energy altered when exposed to external electromagnetic fields, as expressed by $\Delta G_{\mathrm{CT}}^{\mathrm{NEOF}}$ below.

$$\Delta G_{CT}^{NEOF}(p,F) = \Delta G_{CT}^{0} + \delta G_{CT}^{IDI}(p) - \Delta\vec{\mu}.\vec{F} \qquad \text{(SI-29)}$$

where $\Delta G_{\mathrm{CT}}^{0}$ is the intrinsic Gibb free energy gain of the CT state and is independent of both optical field and image dipole interactions. $\delta G_{\mathrm{CT}}^{\mathrm{IDI}}$ is a perturbation associated with the image potentials of CT state dipoles in nearby CNSs. It is a function of the CNS's number of pair but it is in first order spectrally independent of any incident photoexcitation.

The optical field, *F*, is the internal electric field and is related to the external field altered to take into account the polarisation of the semiconductor film through the local field correction, $f = F/F_{\mathrm{ext}}$. $\Delta\vec{\mu}$ is the difference between the dipole moment vectors in the initial and final charge-transfer states.

Assuming that

a) the donor excited and ground states do not carry any dipole moment,

b) λ_{CT} and V_{AD} are both independent of the optical field, and

c) the optical field contribution to the Gibbs free energy is a perturbation,

the decay rate equation can be expanded as Taylor series. An expansion to the 2nd order gives the following expression:

$$k_{CT}^{NEOF} \approx k_{CT}^{IDI}.\left(1 + \delta_{F1}F + \delta_{F2}F^2\right) \qquad \text{(SI-30a)}$$

$$\tau_{CT}^{NEOF} \approx \tau_{CT}^{IDI}.\left(1 - \delta_{F1}F - \delta_{F2}F^2\right) \qquad \text{(SI-30b)}$$

Only 1st and 2nd orders in optical field *F* are considered. The contribution of the image dipole interaction is included in

$$k_{CT}^{IDI} = \left(\frac{4\pi^3}{h^2\lambda_{CT}^0 k_B T}\right)^{1/2} |V_{DA}|^2 exp\left(-\frac{{G_{\lambda-CT}^{IDI}}^2}{4\lambda_{CT}^0 k_B T}\right) \qquad \text{(SI-31a)}$$

We set $G_{\lambda-\mathrm{CT}}^{\mathrm{IDI}} = \Delta G_{\mathrm{CT}}^{0} + \lambda_{\mathrm{CT}}^{0} + \delta G_{\mathrm{CT}}^{\mathrm{IDI}} = G_{\lambda-\mathrm{CT}}^{0} + \delta G_{\mathrm{CT}}^{\mathrm{IDI}}$ with $G_{\lambda-\mathrm{CT}}^{0} = \Delta G_{\mathrm{CT}}^{0} + \lambda_{\mathrm{CT}}^{0}$ to simplify the notations, and we note that the impact of the optical field on the reorganisation energy is explored in the following section, and the expression above can be equivalently written as

$$k_{CT}^{IDI} \approx k_{CT}^{0}.\, exp\left(-\frac{2G_{\lambda-CT}^{0}\delta G_{CT}^{IDI} + {\delta G_{CT}^{IDI}}^2}{4\lambda_{CT}^0 k_B T}\right) \qquad \text{(SI-31b)}$$

$$k_{CT}^{0} = \left(\frac{4\pi^3}{h^2\lambda_{CT}^0 k_B T}\right)^{1/2} |V_{DA}|^2 exp\left(-\frac{{G_{\lambda-CT}^{0}}^2}{4\lambda_{CT}^0 k_B T}\right) \qquad \text{(SI-31c)}$$

$$k_{CT}^{0} = \left(\frac{4\pi^3}{h^2\lambda_{CT}^0 k_B T}\right)^{1/2} |V_{DA}|^2 exp\left(-\frac{{\Delta G_{CT}^{*0}}^2}{k_B T}\right) \qquad \text{(SI-31d)}$$

The superscript "0" indicates that the parameters are altered by neither IDI, nor optical field effects. In eq. SI-30, the linear and quadratic coefficients of the optical field, *F*, terms are defined as:

$$\delta_{F1} = \frac{1}{2\lambda_{CT}^0 k_B T}\frac{G_{\lambda-CT}^{IDI}}{\lambda_{CT}^0}\Delta\mu.\, cos\theta \qquad \text{(SI-32a)}$$

$$\delta_{F2} = \left[\frac{1}{2}\left(\frac{G_{\lambda-CT}^{IDI}}{2\lambda_{CT}^0 k_B T}\right)^2 - \frac{1}{4\lambda_{CT}^0 k_B T}\right].\left(\Delta\mu.\, cos\theta\right)^2 \qquad \text{(SI-32b)}$$

with θ the angle between the CT dipole and the optical field. Its average over all the dipole orientations leads to $\langle\delta_{F1}\rangle = 0$ and to the optical field intensity dependence

$$\langle\delta_{F2}\rangle = \left(\frac{{G_{\lambda-CT}^{IDI}}^2}{2\lambda_{CT}^0 k_B T} - 1\right).\frac{(\Delta\mu)^2}{8\lambda_{CT}^0 k_B T} \qquad \text{(SI-32c)}$$

Figure 3|a shows on the same graph the integral of the optical field intensity calculated at 725 nm across D:A films, $|\Delta F|^2_{\mathrm{intg.}}$ and the CTD experimental data both as a function of the spacer thickness. The calculation presents oscillations of comparable periods as the experimental data points. The maxima of $|\Delta F|^2_{\mathrm{intg.}}$ are relatively well matched with those of CR, while the modulations of CS and CR are out-of-phase as mentioned earlier. Figure 3b presents the experimental CTD data as a function of the difference between the CNS and fused silica substrates optical field intensity. The CTDs measured on 4p CNSs present an almost linear variation with the optical field intensity. The agreement is better for CS than for CR. Noticeably, the slope is negative with CS, whereas CR presents a positive slope. The latter is consistent with the fact that CR is located near the barrierless point,[18] where $G_{\lambda-\mathrm{CT}}^{0} \approx 0$ which consequently satisfies the following inequality,

$$\left|G_{\lambda-CT}^{IDI}\right| < \sqrt{2\lambda_{CT}^0 k_B T} \qquad \text{(SI-33a)}$$

Based on section SI-V.3 and neglecting the triphenylene moieties, with the room temperature thermal energy value of ~25 meV and the total reorganisation energy of perylene ~0.11 eV as estimated when embedded inside a cluster,[48] we note that $\left|G_{\lambda-\mathrm{CT}}^{\mathrm{IDI}}\right| > \sqrt{2\lambda_{\mathrm{CT}}^0 k_B T}$ leads to $|\Delta G_0| > 75$ meV. The coefficient $\langle\delta_{F2}\rangle$ is then negative, resulting in the positive slope of CR characteristic time. For CS characteristic time to decrease with the optical field intensity, then $\langle\delta_{F2}\rangle$ is positive, implying that

$$\left|G_{\lambda-CT}^{IDI}\right| > \sqrt{2\lambda_{CT}^0 k_B T} \qquad \text{(SI-33b)}$$

SI-Figure 11 |a presents the IDI and optical field effects on CT rate parabola, when only the *ΔG* is affected by the optical field. The IDI main effect is to push the system away from the barrierless point. This induces a reduction of the CT rate, i.e. slowdown of the CTDs.[18] In contrast, as F increases $\Delta|\mathrm{F}|^2_{\mathrm{intg}} > 0$, CS gets closer to the barrier-less point, while CR is pushed away from it, resulting in an acceleration and slowdown, respectively. We note that while the IDI effect is dependent on CS and CR being in the normal and inverted region, the optical field effect is determined by the inequalities SI-33a and SI-33b regardless of the Marcus region where the effect occurs. As illustrated by the central vertical dotted lines in SI-Figure 11 |a, the optical field behaviour transition is set by

$$\beta = \sqrt{\frac{2k_B T}{\lambda_{CT}^0}} \qquad \text{(SI-33c)}$$

As CS and CR have different reorganisation energy, we note that the transition is a function of the CT under consideration. However,

tuning the measurement temperature might allow establishing experimentally the scale of the reorganisation energy in the solid-state thin films.

SI-Figure 11 |b presents the IDI and optical field CT alteration mechanisms on Marcus energy diagrams. CS corresponds to transfers from the black curve D*:A (S_1) to those on the upper-right hand-side. CS activation barrier increases when the D:A transition is located in the normal region and is exposed to the IDI effect ; it is consistent with a slowdown of the CS characteristic time with the number of pairs, i.e. a stronger IDI effect. Consequently, the IDI effect can be seen as moving the CT parabola upwards, as illustrated by the displacement between the pink (CT^0) and the blue parabola (CT^{IDI}). Noticeably, this does not affect the reorganisation energy. Furthermore, this upwards shift is also consistent with CR

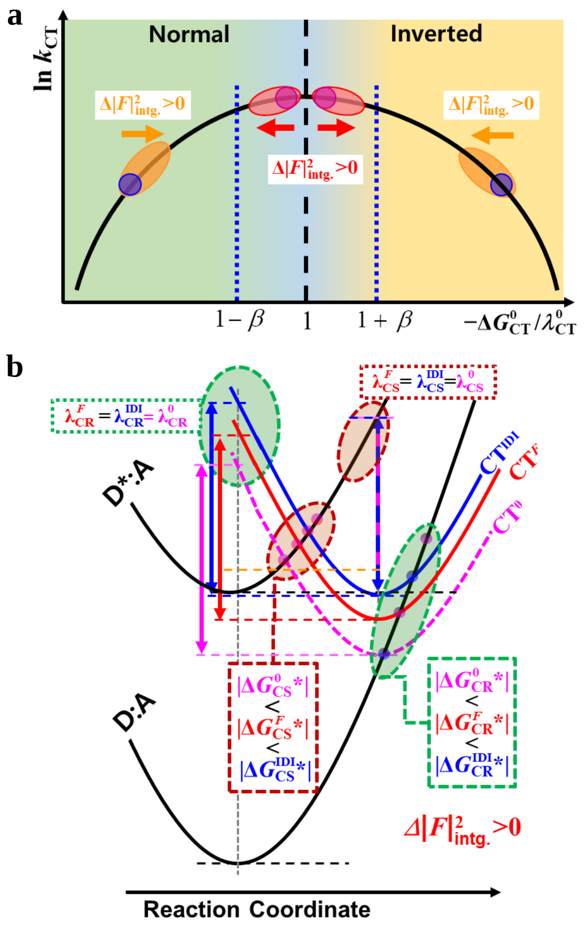

SI-Figure 11 | Marcus Energy diagrams when only the driving force is affected by optical fields. a, Dependence of the logarithm of the CT rates with the intrinsic driving force ($-\Delta G^0_{CT}$) normalised with the intrinsic reorganisation energy (λ^0_{CT}). The blue area is the barrierless region. The blue dots correspond to the CT rates subjected to IDI effects. The ellipses illustrate the effect of the optical field intensity ($\Delta|F|^2_{intg}$). The arrow directions indicate the impact on $-\Delta G^0_{CT}/\lambda^0_{CT}$ and k_{CT} of the optical field. **b,** Potential energy surfaces as a function of the reagent and product nuclear coordinates. D:A and D*:A are the ground (S_0) and first excited (S_1) states of the donor, respectively. CT^x are the charge transfer states of the D:A on standard fused silica surface (*x*: 0), CNSs without (*x*: IDI) and with (*x*: *F*) the optical field taken into account, respectively. The coloured dots inside the brown and green ellipses are associated with the CT activation energies (ΔG^{x*}_{CT}) of CS and CR, respectively. The vertical coloured arrows illustrate the variation of the reorganisation energy (λ^x_{CT}). An upwards (downwards) displacement of the CT Marcus parabola under the effect of an increase of the optical field intensity ($\Delta|F|^2_{intg}>0$) would lead to an increase (decrease) of the activation energies for both CS and CR, which would then be in-phase.

being in the inverted region. Then, its activation barrier increases under the IDI effect, which is consistent with a slowdown of CR characteristic time when IDI effect increases.

In the present system, CR corresponds to an electron transfer from the upper-right hand-sides CT parabolas to the black one, labelled D:A and corresponding to ground state (S_0) of the system.

Still, when the effect of the optical field on the reorganisation energy is neglected and only eq. SI-29 is taken into account, SI-Figure 11 |b shows that the effect of the optical field would also be to move the CT parabola vertically. Let's focus on the case $\Delta|F|^2_{\text{intg}}>0$. The CT energy parabola would go down (red parabola), which in terms of activation energy would be consistent with the behaviour of CS shown in Figure 3b. For CR, however, a downward movement of the CT parabola would also lead to a smaller activation energy under the influence of $\Delta|F|^2_{\text{intg}}>0$ when compared with the IDI effect alone. However, this would not be consistent with the experimental data presented in Figure 3b. Assuming that $\Delta|F|^2_{\text{intg}}>0$ would move the CT parabola upwards would match with CR experimental data but would be inconsistent with CS data. For sake of clarity, this case is not represented in SI-Figure 11 |.

Consequently, a simple upward-downwards shift of the CT parabola under the influence of $|F|^2$ cannot explain the out-of-phase CS and CR dynamics. It is obvious that a more complex shift of the CT parabola has to be considered. We should then take into account CT parabola horizontal movements, i.e. reorganisation energy should be affected by optical field. This is consistent with reports on OPV D:A molecular systems suggesting that external reorganisation energy can be affected by a static external electric field.[99-102] Their numerical simulations force us to question and, to eventually, reconsider the limitation of a situation where the optical field would affect the driving force only.

SI-V.4.b. Optical Effect on the Reorganisation Energy (λ)

In the above-mentioned literature,[99-102] DFT calculations show that the inner reorganisation energy is much less affected than the external reorganisation by the application of an external electric field. The latter varies linearly with the external field, as expressed below

$$\Delta\lambda_{env}=\lambda_{env}(F)-\lambda_{env}(0)=-\Delta\vec{\mu}.\vec{F} \qquad \text{(SI-34a)}$$

Thanks to eq. SI-26, it is can also be written to match the presentation of the optical field on the Gibbs free energy given in eq. SI-29 and to obtain the expression of the reorganisation energy taken into account the effect of the optical field

$$\lambda^{NEOF}_{CT}(F)=\lambda^0_{CT}-\Delta\vec{\mu}.\vec{F} \qquad \text{(SI-34b)}$$

with λ^0_{CT} the intrinsic reorganisation energy of the D:A molecules in the thin films.

Let's then assume that

a) The donor excited and ground states do not carry any dipole moment,
b) ΔG, V_{AD} and the inner reorganisation energy are independent of the optical field, and
c) the optical field contribution to the reorganisation energy is a perturbation, which has then the same amplitude as the one used previously for the Gibbs free energy gain.

The decay rate equation can then be again expanded as Taylor series leading to a similar equation as eq. SI-30, with the following coefficients

$$\delta_{F1}=\left[\frac{1}{4\lambda^0_{CT}k_BT}\frac{G^{IDI}_{\lambda-CT}}{\lambda^0_{CT}}\left(2-\frac{G^{IDI}_{\lambda-CT}}{\lambda^0_{CT}}\right)+\frac{2}{\lambda^0_{CT}}\right]\Delta\mu.cos\theta \qquad \text{(SI-34c)}$$

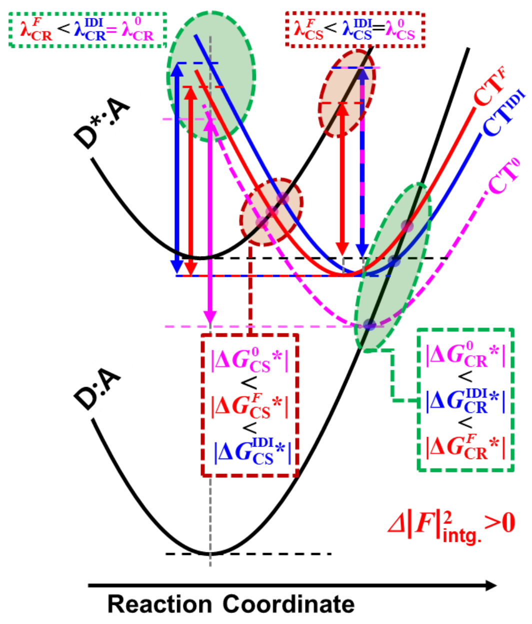

SI-Figure 12 | Marcus energy diagrams when only the reorganisation energy is affected by optical fields. Profile of potential energy surfaces as a function of the reagent and product nuclear coordinates. D:A and D*:A are the ground (S_0) and first excited (S_1) states of the donor, while CT^x are the charge transfer states of the D:A on standard (x: Al_2O_3), CNSs without (x: IDI) and with (x: *F*) the optical field taken into account, respectively. The coloured dots inside the green ellipses are associated with the CT activation energies ($|\Delta G^*|$), while the coloured arrows illustrate the variation of the reorganisation energy.

$$\begin{aligned}
&\delta_{F2} \\
&= \left\{ \frac{3}{(2\lambda^0_{CT})^2} + \left(\frac{{G^{IDI}_{\lambda-CT}}^2}{2\lambda^0_{CT} k_B T} - 1 \right) \frac{1}{4\lambda^0_{CT} k_B T} \right. \\
&+ \frac{G^{IDI}_{\lambda-CT}}{\lambda^0_{CT}} \left\{ \frac{1}{4\lambda^0_{CT} k_B T} \left(1 + \frac{1}{2\lambda^0_{CT} k_B T} \right) \right. \\
&+ \frac{G^{IDI}_{\lambda-CT}}{\lambda^0_{CT}} \left\{ \frac{-3}{16\lambda^0_{CT} k_B T} \right. \\
&+ \frac{G^{IDI}_{\lambda-CT}}{\lambda^0_{CT}} \left[\frac{-2}{(4k_B T)^2} \right. \\
&\left.\left.\left.\left. + \frac{G^{IDI}_{\lambda-CT}}{\lambda^0_{CT}} \frac{1}{2(4k_B T)^2} \right] \right\} \right\} \right\} . (\Delta\mu . cos\theta)^2
\end{aligned} \quad \text{(SI-34d)}$$

As previously stated, averaging over all the dipole orientations leads to $\langle\delta_{F1}\rangle = 0$ and to the optical field intensity dependence

$$\langle\delta_{F2}\rangle = \frac{1}{2}\delta_{F2}(\theta = 0) \quad \text{(SI-34e)}$$

Whilst eq. SI-34d is more complex than when the driving force alone is considered, we can identify the components resulting from the reorganisation energy term contained in the numerator of the activation energy, which directly echo eq. SI-32c. The $\langle\delta_{F2}\rangle$ coefficient still justifies the fact that in Figure 3a, the integral of the optical field intensity across D:A films, $\Delta|F|^2_{\text{intg}}$, and the CTD experimental data points present comparable oscillations. Natu-rally, establishing the optical field behaviour transition cannot be set anymore by the parameter β given in eq. SI-33c but it will still occur when $\langle\delta_{F2}\rangle$ equals zero and changes sign. At the barrierless point, $G^{\text{IDI}}_{\lambda-\text{CT}} = 0$ and $\langle\delta_{F2}\rangle$ is dominated by the thermal agitation and is negative. This is consistent with the CS characteristic time data presenting a positive slope as illustrated in Figure 3b.

The discussion having led to the conclusion that the optical field effect is to shift horizontally the CT energy parabola on SI-Figure 11 |remains valid. It is consistent with the expected variation of the reorganisation energy and is schematically presented in SI-Figure 12 |. A horizontal shift of the CT parabola was chosen to the right only for illustration purposes. It shows that a decrease of the activation energy of CS corresponds to an increase of the activation energy of CR. A shift to the left of the CT parabola would then correspond to an increase of CS activation energy and a decrease of CR activation energy. The out-of-phase behavior of CS and CR can then be reproduced qualitatively by taking into account the fact that the reorganisation is affected by the optical field.

SI-V.4.c. Optical Effects on ΔG and λ

Obviously, beyond the validity check of considering each parameter separately, one should not ignore that both driving force (eq. SI-28) and reorganisation energy (eq. SI-34b) are affected by the optical field. This situation is explored in this section.

As stated earlier, the nonlocal enhanced optical field (NEOF) effect can be written for the Gibbs free energy gain and the reorganisation energy through eqs. SI-29 and SI-34b, respectively.

$$\Delta G^{NEOF}_{CT}(p, F) = \Delta G^0_{CT} + \delta G^{IDI}_{CT}(p) - \Delta\vec{\mu}.\vec{F} \quad \text{(SI-29)}$$

$$\lambda^{NEOF}_{CT}(F) = \lambda^0_{CT} - \Delta\vec{\mu}.\vec{F} \quad \text{(SI-34b)}$$

At this stage, we acknowledge that we cannot yet question whether or not optical field effects have exactly the same amplitude on both the Gibbs free energy gain and the reorganisation energy of the environment. However, we stress that by generalizing Marcus theory with NEOF effect only aims at a qualitative agreement between the theoretical description and the experimental data, so that the above-mentioned question is definitely beyond the scope of the present work.

Nonetheless, let's again assume that

a) the donor excited and ground states do not carry any dipole moment,
b) V_{AD} and the inner reorganisation energy are independent of the optical field and
c) both the IDI and optical field effects are perturbations.

Then, the decay rate equation can be expanded as Taylor series, which to the 2nd order gives the following expressions:

$$\delta_{F1} = \left[\frac{1}{\lambda^0_{CT} k_B T} \frac{G^{IDI}_{\lambda-CT}}{\lambda^0_{CT}} \left(2 - \frac{G^{IDI}_{\lambda-CT}}{\lambda^0_{CT}} \right) + \frac{2}{\lambda^0_{CT}} \right] \Delta\mu . cos\theta \quad \text{(SI-35a)}$$

$$\begin{aligned}
&\delta_{F2} \\
&= \left\{ \frac{3}{(2\lambda^0_{CT})^2} + \frac{1}{\lambda^0_{CT} k_B T} \left(\frac{{G^{IDI}_{\lambda-CT}}^2}{4\lambda^0_{CT} k_B T} - 1 \right) \right. \\
&+ \frac{G^{IDI}_{\lambda-CT}}{\lambda^0_{CT}} \left\{ \frac{5}{4\lambda^0_{CT} k_B T} \right. \\
&+ \frac{G^{IDI}_{\lambda-CT}}{\lambda^0_{CT}} \left\{ \frac{-3}{16\lambda^0_{CT} k_B T} \right. \\
&+ \frac{G^{IDI}_{\lambda-CT}}{\lambda^0_{CT}} \left[\frac{-2}{(4k_B T)^2} \right. \\
&\left.\left.\left.\left. + \frac{G^{IDI}_{\lambda-CT}}{\lambda^0_{CT}} \frac{1}{(4k_B T)^2} \right] \right\} \right\} \right\} . (\Delta\mu . cos\theta)^2
\end{aligned} \quad \text{(SI-35b)}$$

Again, averaging over all the dipole orientations leads to $\delta_{F1} = 0$, and to the optical field intensity dependence

$$\langle\delta_{F2}\rangle = \frac{1}{2}\delta_{F2}(\theta = 0) \quad \text{(SI-35c)}$$

Noticeably, equations eq. SI-29 and eq. SI-34b are linked through the linear relation presented below.

$$\Delta G^{NEOF}_{CT}(p, F) = \Delta G^0_{CT} - \lambda^0_{CT} + \delta G^{IDI}_{CT}(p) + \lambda^{NEOF}_{CT}(F) \quad \text{(SI-36)}$$

In addition, the mathematical expression of Marcus equation obtained when starting from eq. SI-19 and developed with both eq. SI-29 and eq. SI-34c leads to a similar result as the development based on just eq. SI-34c. The main difference is that the generalised

activation energy $\Delta G_{\mathrm{CT}}^{\mathrm{NEOF}*}$ obtained from eq. SI-20 includes $-2\Delta\vec{\mu}.\vec{F}$, instead of $-\Delta\vec{\mu}.\vec{F}$, in the numerator as presented in the equations below.

$$\Delta G_{CT}^{*NEOF} = \frac{\left(\lambda_{CT}^{0} + \Delta G_{CT}^{0} + \delta G_{CT}^{IDI}(p) - 2\Delta\vec{\mu}.\vec{F}\right)^{2}}{4\left(\lambda_{CT}^{0} - \Delta\vec{\mu}.\vec{F}\right)} \quad \text{(SI-37a)}$$

$$\Delta G_{CT}^{*NEOF} \approx \frac{\left(\lambda_{CT}^{0} + \Delta G_{CT}^{0} + \delta G_{CT}^{IDI}(p) - 2\Delta\vec{\mu}.\vec{F}\right)^{2}}{4\lambda_{CT}^{0}} \quad \text{(SI-37b)}$$

SI-V.5. Schematic Representations of Nonlocal Enhanced Optical Field Effects in Generalised Marcus Theory

SI-V.5.a. Potential Energy Surfaces

SI-Figure 13 | presents the experimental CTD data plotted as a function of the difference between the CNS and FS substrates optical field intensity at the probe beam wavelength of 725 nm. Its content is identical to Figure 3b, but distributed on two distinct panels, one for CS and the other for CR on SI-Figure 13 |a and b, respectively. Fused silica supports no IDI contribution and there consequently is a negligible optical field effect, CTD is then the fastest for both CS and CR (pink data point labelled “0”). Noticeably, when a 4p CNS with a 150 nm Al_2O_3 is used to spincoat the D:A thin films, the resulting optical field is similar to the one obtained on FS ($\Delta|F|_{\mathrm{intg}}^{2} \cong 0$) but the CTD data increases drastically following the vertical “pink to blue” arrows to reach the slower CDT labelled “1” in blue.

With partially destructive interferences between incident and reflected optical beams, the amplitude of the optical field intensity is decreased ($\Delta|F|_{\mathrm{intg}}^{2} < 0$), and the CTD follows the “horizontal blue to orange” line with an orientation towards the left hand-side

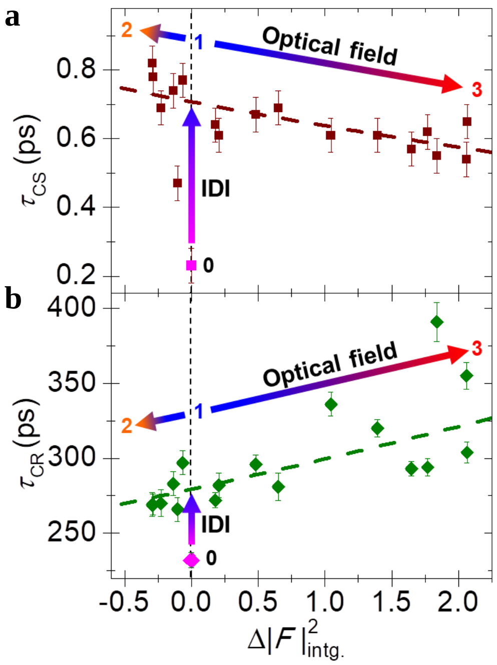

SI-Figure 13 | Analysis of nanophotonic control over CTDs by nonlocal enhanced optical field effects. Linear variation of a, CS (■) and b, CR (♦) dynamics with the integral difference of optical field intensity between CNs and fused silica. The bottom pink data points were obtained on fused silica, the vertical and horizontal arrows materialise IDI nonlocal effect and IDI enhanced optical field effects, respectively. These are echoed by the colour code (pink to blue, blue to orange, blue to red) and labelling (0, 1 and 2-3) indicating the reference data point on FS, the IDI effect on 4p CNSs, the negative and the positive $\Delta|F|_{\mathrm{intg}}^{2}$ variation of the nonlocal enhanced optical effect, respectively. Dashed lines are guide-for-the-eyes. The optical field intensity was determined for a 725 nm incident beam and integrated across the organic D:A thin films on top of 4p CNSs.

of SI-Figure 13 | to reach the CDT data points labelled “2” and stated in orange for consistency purposes. In SI-Figure 13 |a, CS characteristic times are increased indicating a slower charge separation. CS and CR dynamics are out-of-phase, and decreasing the optical field intensity across the D:A film induces a decrease of the CR characteristic times, indicating a faster charge recombination when the optical field intensity is reduced. The opposite effect occurs when the optical field difference is increase ($\Delta|F|_{\mathrm{intg}}^{2} > 0$). With constructive interferences between incident and reflected optical beams, and the CTD follows the “horizontal” blue to red line with an orientation towards the right hand-side of SI-Figure 13 |. The arrows reach the CTD data points labelled “3” stated in red. In SI-Figure 13 |a, CS characteristic times are decreased indicating a faster charge separation. CR being out-of-phase, increasing the optical field intensity across the D:A film leads the CR characteristic times to increase, indicating a slower charge recombination when the contribution of the optical field intensity is increased.

We note that $\Delta|F|_{\mathrm{intg}}^{2} \cong 0$ implies only that the optical field intensities in “0” and “1” are comparable. However, we stress that beyond the convenience of using the optical field intensity across the D:A thin film spread on fused silica, this choice does not mean that the IDI and optical field effects are uncorrelated in any of the CNS herein presented. On the contrary, we have shown in the theoretical section that IDI and optical field effects are very much correlated with one another, as the former enhances the latter.

On top of FS, data points “0”, there are strictly no IDI effects, however, a very weak optical field effect could eventually be observed ; even if we stress that it would smaller than the resolution of our experimental setup.

The blue points labelled “1”, for which $\Delta|F|_{\mathrm{intg}}^{2} \cong 0$, correspond to a CNSs supporting IDI and to $|F|_{\mathrm{intg}}^{2} \neq 0$. In these structures, IDI and optical field effects are already correlated. Nonetheless, even for the smallest $|F|_{\mathrm{intg}}^{2}$ value on the very left hand-side of SI-Figure 13 |, the CTD alterations result from the nonlocal enhanced optical field (NEOF) effect and not from a single IDI effect. This situation is nonetheless the experimental condition presenting the weakest correlations, hence the strongest IDI relative contribution. In this case, IDI is still shown to induce a slower CTD for both charge separation and recombination when compared with the pink FS data points, which again do not support any IDI effect.

The CNS experimental CTD data can be represented on a Marcus energy diagrams such as those presented in the SI-Figure 14 |. The convention herein adopted is the same as in SI-Figure 11 | and SI-Figure 12 |, where CS corresponds to transfers from the black curve D*:A (S_1) to those on the upper-right hand-side (CT), and CR to transfers from the upper-right hand-side parabola (CT) to the black curve D:A (S_0). The colour code of the CT parabola is the same in SI-Figure 13 |, pink for the FS reference, blue under the IDI effect, while the NEOF effect is represented in orange and red when $\Delta|F|_{\mathrm{intg}}^{2}$ is negative and positive, respectively. We have shown that IDI induces an upper shift, without any change of the reorganisation energy, whereas NEOF should combine both vertical and horizontal shifts of the CT parabola. The question is then to determine which combination would be consistent with the CTD data of the D:A system considered in this work.

Based on the out-of-phase CTD data the only CT parabola shifts that would satisfy the information stated on SI-Figure 13 | would be $\Delta|F|_{\mathrm{intg}}^{2} < 0$ and $\Delta|F|_{\mathrm{intg}}^{2} > 0$ leading to upwards-left and downwards-right, respectively, as illustrated in SI-Figure 14 |.

SI-Figure 14 |a illustrates the case of a decrease of the optical field. As shown in the right hand-side schematic, the CT parabola is moved upwards and to the left hand side simultaneously. The upper brown box located in the middle of SI-Figure 14 |a focusses on CS, while the lower green box focusses on CR. On the very

right-hand side, we present the associated relative variations of the activation energy ($\Delta G_{\mathrm{CT}}^{\mathrm{x}*}$), Gibbs free energy gain ($\Delta G_{\mathrm{CT}}^{\mathrm{x}}$, driving force) and reorganisation energy ($\lambda_{\mathrm{CT}}^{\mathrm{x}}$).

SI-Figure 14 |b is built similarly to illustrates the case of an increase of the optical field. As shown in the right hand-side schematic, the CT parabola is moved downwards and to the right-hand side simultaneously. Again, the upper brown box and the lower green box focus on CS and CR, respectively. The very right-hand side presents the relative variations of $\Delta G_{\mathrm{CT}}^{\mathrm{x}*}$, $\Delta G_{\mathrm{CT}}^{\mathrm{x}}$ and $\lambda_{\mathrm{CT}}^{\mathrm{x}}$.

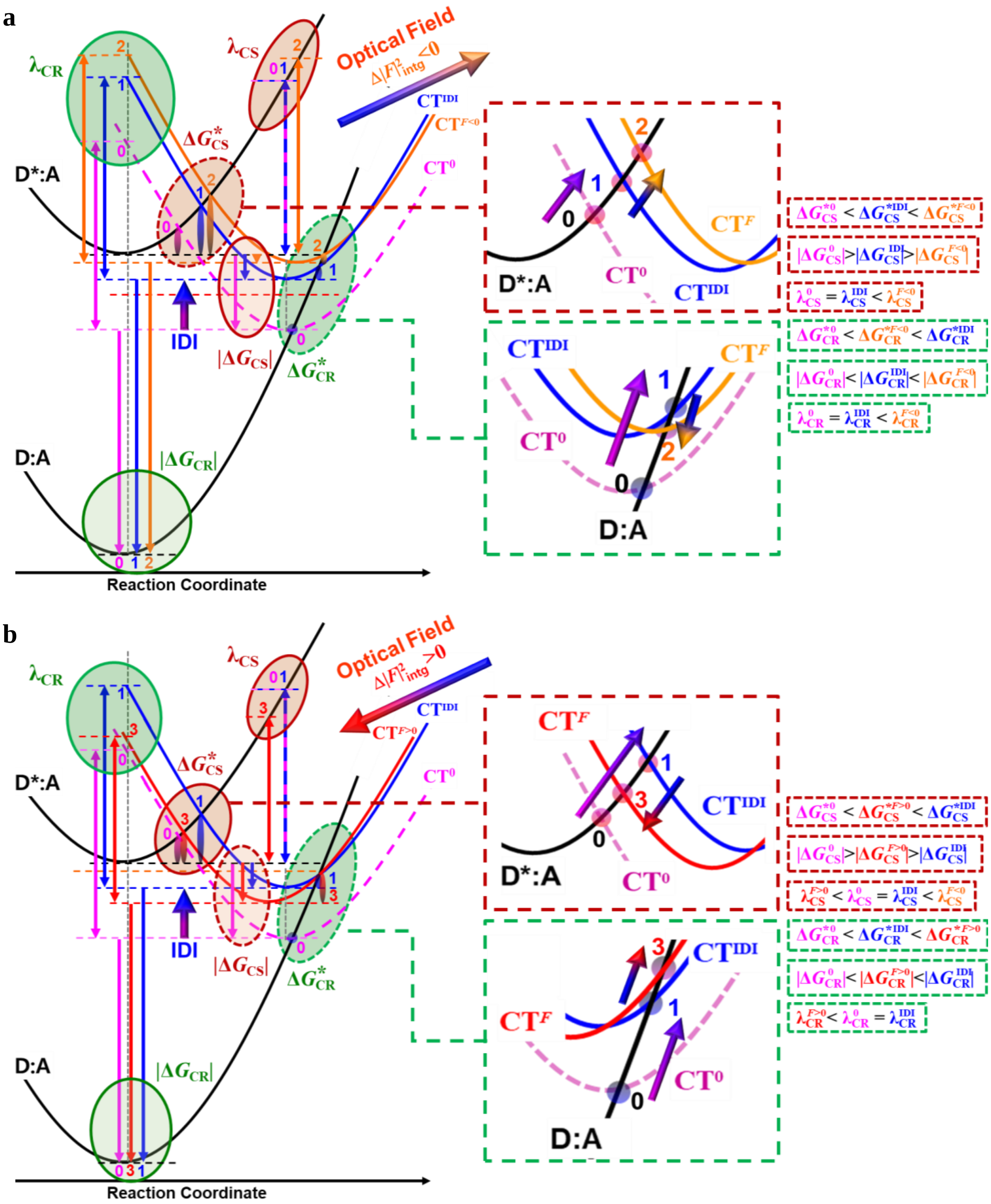

SI-Figure 14 | Marcus energy diagrams under the influence of NEOF effects. Potential energy surfaces as a function of the reagent and product nuclear coordinates for **a,** $\Delta|F|_{\mathrm{intg}}^{2} < 0$ (upper right shift) and **b,** $\Delta|F|_{\mathrm{intg}}^{2} > 0$ (lower left shift). Left hand-side, middle and left hand-side present the whole potential energy surfaces, close-up views on the activation energy, evolution of the CT activation energy ($\Delta G_{\mathrm{CT}}^{\mathrm{x}*}$), Gibbs free energy gain ($\Delta G_{\mathrm{CT}}^{\mathrm{x}}$, driving force) and reorganisation energy ($\lambda_{\mathrm{CT}}^{\mathrm{x}}$) with the effects, respectively. D:A and D*:A are the ground (S_0) and first excited (S_1) states of the donor, respectively. CT^{x} are the charge transfer states of the D:A on standard fused silica surface (x: 0), CNSs without (x: IDI) and with (x: F) the optical field effect taken into account, respectively. The coloured dots inside the brown and green ellipses are associated with the $\Delta G_{\mathrm{CT}}^{\mathrm{x}*}$ of CS and CR, respectively. The upper and lower vertical coloured arrows illustrate the variations of $\lambda_{\mathrm{CT}}^{\mathrm{x}}$ and $\Delta G_{\mathrm{CT}}^{\mathrm{x}}$, respectively.

To summarise, optical fields affect both driving force and reorganisation energy, and consequently they should be inserted in a generalised formalism aiming at calculating the CT rates. This is justified both by the analysis consistency herein presented, and by earlier works having explored the influence of static electric field on both driving force and reorganisation energy should be affected. The rational of this approach is further strengthened by considering that external optical fields should definitely impact on the electronic energy level as much as on the nuclear rearrangement, which are associated with driving force and reorganisation energy, respectively. In addition, we stress that the nonlocal enhanced optical field effect, herein reported on, is directly linked with image dipole interactions supported by the nanophotonic structures on which the D:A thin films were spincoated. Without the IDI effect, the impact of the optical field on the CTDs would remain negligible and would probably not have been observed.

SI-V.5.b. Dependence of the Logarithm of the CT Rates

SI-Figure 15 | presents the calculated logarithm of the CT rates as a function of the driving force and the reorganisation energy. Typical cases echoing Figure 4a as well as the sections SI-V.1 and SI-V.4 above have been selected to illustrate the relative impacts of the IDI and NEOF perturbations.

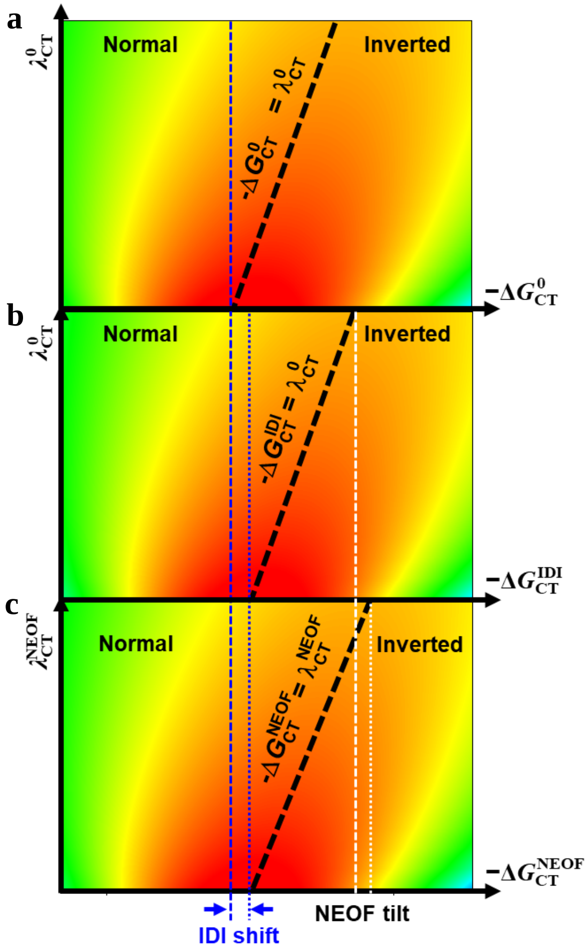

SI-Figure 15 | False-colour plots of the CT rate logarithm calculated as a function of a, the intrinsic driving force ($-\Delta G^{0}_{CT}$) and the intrinsic reorganisation energy (λ^{0}_{CT}), **b,** the IDI altered driving force ($-\Delta G^{IDI}_{CT}$) and the intrinsic reorganisation energy (λ^{0}_{CT}), **c,** the NEOF altered driving force ($-\Delta G^{NEOF}_{CT}$) and the NEOF altered reorganisation energy (λ^{NEOF}_{CT}). Normal and inverted regions are on the left and right hand-sides of the figure, respectively, and separated by the tilted black dashed lines corresponding to the barrierless zone. The vertical blue lines mark the borderless zone shift under an IDI perturbation. The white lines emphasise the tilt of the borderless zone under the influence of the optical field effect applied to both the driving force and the reorganisation energy.

SI-Figure 15 |a is a typical result of calculations completed based on eq. SI-19 for intrinsic driving force and reorganisation energy. The normal and inverted regions are separated by the barrierless zone, corresponding to the maxima of the CT rates, which is represented by a tilted black dashed line and obtained when $\Delta G^{0}_{CT} + \lambda^{0}_{CT} = 0$.

SI-Figure 15 |b considers the image dipole interaction effect which only applies to the driving force, $\Delta G^{IDI}_{CT} = \Delta G^{0}_{CT} + \delta G^{IDI}_{CT}$, while the reorganisation energy remains unaffected. The IDI effect shifts the barrierless zone by the δG^{IDI}_{CT} value corresponding to eq. SI-21 and SI-18. The vertical blue lines in SI-Figure 15 |b illustrate the borderless zone shift under the IDI perturbation. For sake of clarity, only one sign of δG^{IDI}_{CT} is considered.

Finally, SI-Figure 15 |c displays the impact of the nonlocal enhanced optical field effect on both the driving force, $\Delta G^{NEOF}_{CT} = \Delta G^{0}_{CT} + \delta G^{IDI}_{CT} - \Delta\vec{\mu}.\vec{F}$ (eq. SI-29) and the reorganisation energy, $\lambda^{NEOF}_{CT} = \lambda^{0}_{CT} - \Delta\vec{\mu}.\vec{F}$ (eq. SI-34b). To the shift revealed in SI-Figure 15 |b adds a tilt of the barrierless zone, which obviously depends on the sign of the optical field effect. Again, for sake of clarity only one sign of optical field effect is presented. This tilts of the borderless zone under the influence of the optical field effect is materialised by the vertical white lines.

SI-V.6. Comments on the Generalisation Formalism

Regarding the generalized Marcus theory, in the approach we have developed, the optical field can be seen as replacing an electrical bias. However, it is worth considering that whilst applying an external bias could allow for knowing quantitatively what electric field value to insert in Marcus theory, the situation is a lot more complex in the case of optical fields triggering such mediators such as SPVs (regardless of them being influenced by IDI / non-local effects).

SPVs were measured at the top of bare substrates to demonstrate that they exist and are influenced by the morphology of the CNSs. However, the fact of adding an organic top layer certainly affects the photo induced bias at, or close to, the interface of the organic semiconductor and dielectric layers. It is this latter configuration, the bias which ideally would be inserted in the "standard" Marcus theory cannot be easily accessed. Indeed, measuring the SPV at the top of the organic might be slightly related to, but will not give any quantitative value for what the organic layer experiences when in contact with the multilayer structure.

In contrast, the present approach takes advantage of the fact that the optical field can be calculated based on the optical constants, which can be measured independently for each parts of the system. As a consequence, calculating and using the optical field as a parameter to develop the generalized Marcus theory can be both more practical and more relevant to complex structures. In addition, we note that this approach can be applied without any changes in the vicinity of the SPV effect observed on bare CNSs, because the SPV and the optical field are linked. In other words, the generalization part to Marcus theory introduces an electrodynamics component which is usually not in Marcus theory.

SI-VI. Control Experiments and NEOF Analysis Consistency Checks

Now that the theoretical framework has been developed and validated with the 4p CNSs, let's apply it to control experiments, including those presented in section SI-IV.2.

SI-VI.1. Relative Contributions of the Pump and Probe Beams

SI-Figure 16 |a illustrates the sample configuration and the optical beam standard parameters in terms of wavelength, power and diameter. SI-Figure 16 |b presents the reflectance spectrum of Ag-

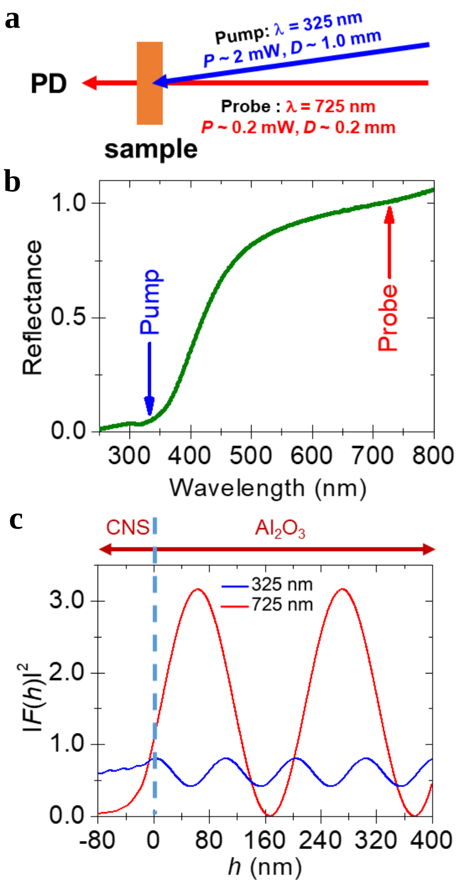

SI-Figure 16 | Pump and probe beam relative contribution to the optical field intensity. a, Schematic of the photodetector (PD), sample and characteristics of the pump and probe beams. **b,** Reflectance of a 4p Ag-Al_2O_3 CNS covered by 10 nm Al_2O_3. c, Optical field intensity distributions of both pump and probe beams across the Al_2O_3 top-covers.

Al_2O_3 CNSs covered with 10 nm Al_2O_3. The arrows point at the reflectance of the substrates at the wavelength of the pump beam (downwards, blue arrow) and probe beam (upwards, red arrow), respectively. The former is much smaller than the latter due to the silver metal layers used to fabricate the substrates. Associated with the respective diameters and powers of the beams, the invariant embedding method was used to calculate the relative reflectance amplitude of each beam. The result is presented in SI-Figure 16 |c with the position of the silver Al_2O_3 thick cover interface marked by the vertical dashed line. The period of the modulation of each beam is obviously very different but more importantly, the optical field intensity of the probe beam, $|F_{725\ nm}|^2$, is about four times larger than the one of the pump beam, $|F_{325\ nm}|^2$. This factor will further increase due to the D:A thin film located on top of the CNS and the triphenylene absorption at 325 nm. Consequently, the influence of the pump beam on the charge transfer dynamics is confirmed to be negligible compared to the probe beam optical field effect.

SI-VI.2. Ruling out Non-Linear Optical Effects

To rule out any non-linear optical effects, SI-Figure 17 | presents unnormalised transient absorption for both CS (SI-Figure 17 |a) and CR (SI-Figure 17 |b). These unnormalised data do not display any obvious amplitude variations with the thickness of the Al_2O_3 cover that could otherwise be related to the CNSs.

To explore this path further, the absolute values of the relative transmission signal variation ($\Delta T/T$), the absolute values of the transmission signal variation (ΔT), and the static 725 nm transmission (T) were extracted and are plotted in SI-Figure 18 |a, b, and c, respectively. In SI-Figure 18 |a and b the absolute values of $\Delta T/T$ and of ΔT are slightly larger for CS than for CR because of the smaller time delay range. These are average values of all the measurements collected to reduce the uncertainty, while the error bars correspond to the experimental standard deviations as obtained from the buffer of Igor collection program. Within the error bars, $|\Delta T/T|$, $|\Delta T|$, and T are relatively constant and they are definitely independent of both the Al_2O_3 thickness and the probe beam optical field intensity across the organic dyad thin film. This also suggest that that no quenching of the CT occurred when the D:A molecules are deposited on top of the CNSs and that only CT dynamics are altered.

Finally, to assess the driving strength this transition was subjected to, we calculated the incident power of the pulsed probe beam on the CNS. These are presented in SI-Figure 19 | for both the absolute values of $\Delta T/T$ and ΔT. Naturally, the transmission signals do not vary with the incident power, which is consistent with SI-Figure 17 | and SI-Figure 18 |. Noticeably, the maximum incident beam peak intensity across the D:A thin film on any CNS is only about half GW/cm^2. This is more than 10 times smaller than typical values used for z-scan measurements, and also smaller than typical powers used optical limiting experiments completed on perilene diimide derivatives.[104-107].

Therefore, these unnormalised data show that the charge dy-

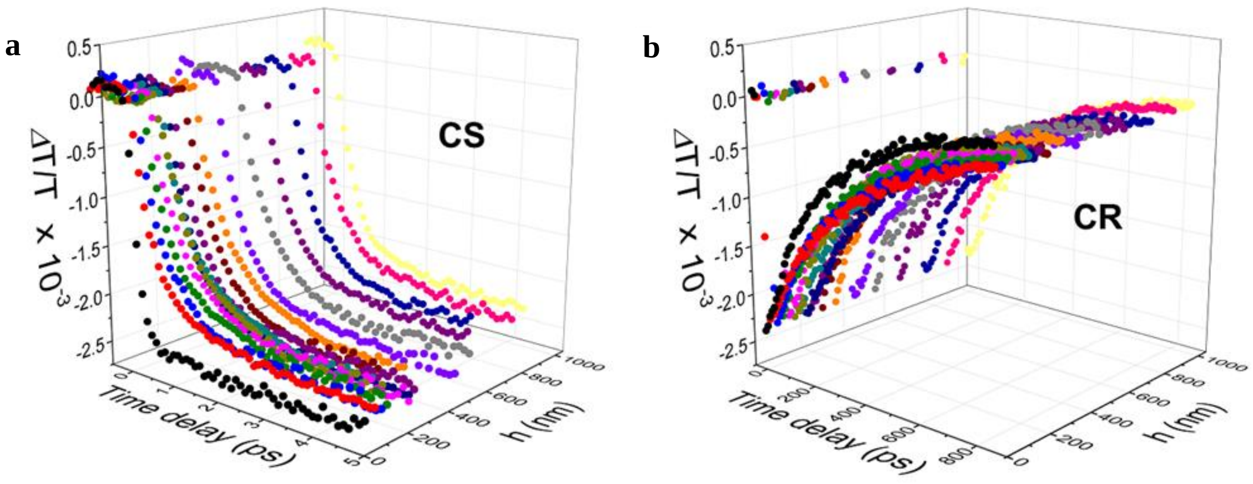

SI-Figure 17 | Unnormalised TA data. a, charge separation and **b,** charge recombination dynamics on a 3D plot: TA signal, time delay and thickness (*h*) of the top Al_2O_3 cover of the CNSs.

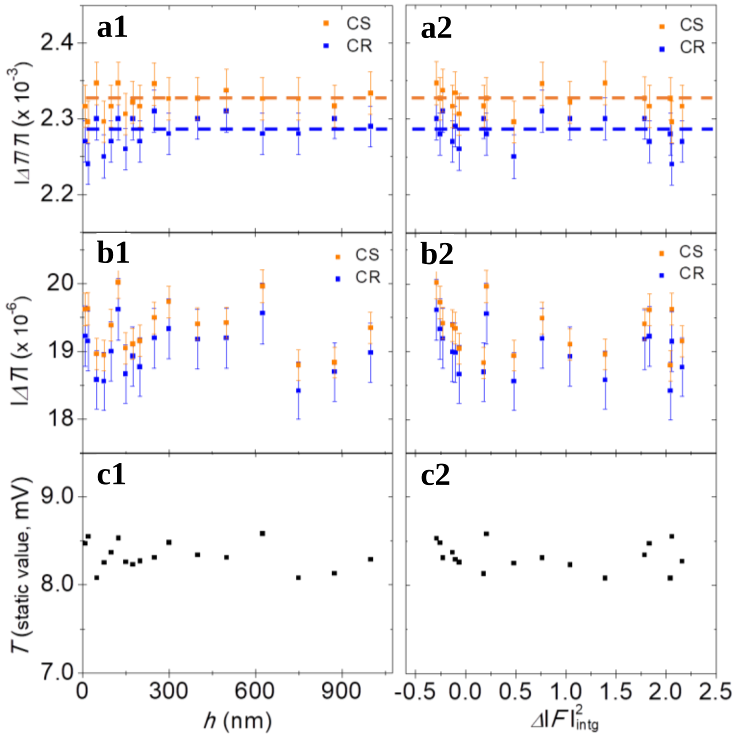

SI-Figure 18 | Unnormalised TA data analysis. a, Absolute values of the relative transmission signal variation ($|\Delta T/T|$). **b,** Absolute values of the transmission signal variation ($|\Delta T|$), and c, the static 725 nm transmission (T) as a function of the oxide layer thickness (1) and the 725 nm optical field variation (2). The dashed lines are guide for the eyes.

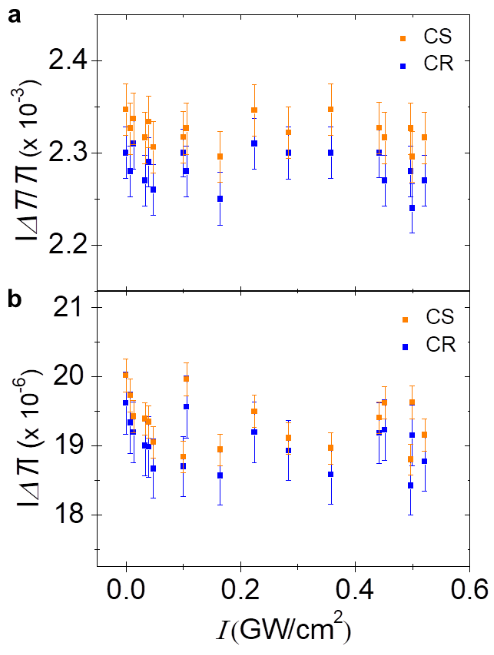

SI-Figure 19 | Optical driving strength. a, Absolute values of the relative transmission signal variation ($|\Delta T/T|$) and **b,** absolute values of the transmission signal variation ($|\Delta T|$) as a function of the 725 nm incident beam peak intensity across the D:A thin film spincoated on-top of the CNSs. The dashed lines are guide for the eyes.

namic measurements completed by transient absorption were not affected by non-linear optical effects, which then could not explain the unusual charge transfer dynamics variation observed in organic semiconductor molecules deposited on CNSs.

SI-VI.3. NanoPhotonic and Thick Metal Films Structures

SI-Figure 20 |a illustrates the modulation of $|F|^2$ with Al_2O_3 on top of both 200 nm thick films of silver and the 4 p CNS. This non-monotonous behaviour is driven by the reflection of the incident beam. It is then natural that the oscillations are qualitatively reproduced on top of both surfaces, even if there is a noticeable phase-shift when comparing the two sets of substrates.

For convenience purposes, SI-Figure 20 |b replots the CTD experimental data as a function of the optical field intensity integrated across the D:A films spin-coated on top the Al_2O_3 covers of selected thicknesses. As mentioned earlier the CS variation on top of the 200 nm thick Ag films is within the error bar of the measurements. Nonetheless, the fit of all the data points suggests a negative slope, which is consistent with the observation made with 4p CNSs. SI-Figure 20 |b1 illustrates the opportunity of the present analysis for both 200 nm thick Ag films and the CNSs.

SI-Figure 20 |b2 shows the CR data obtained with these two structures. The CR data points obtained on-top of 200 nm thick Ag films present a variation, which is larger than the experimental uncertainties. The positive slope resulting from this analysis is consistent with the behaviour observed on top of 4p Ag: Al_2O_3 CNSs.

Noticeably, the 200 nm thick metal film has a much weaker IDI contribution, which leads to slopes that are within and barely larger than the experimental error, for CS and CR, respectively. The slope values are presented in SI-Table 2. The slope amplitude differences are also consistent with the model developed in the sections above.

SI-Table 2. Slope of the charge separation (a_{CS}) and recombination (a_{CR}) times as a function of the integral of the optical field intensity on 200 nm Ag thick films and 4 p CNSs covered with different Al_2O_3 top cover thicknesses.

substrate	a_{CS} (ps)	a_{CR} (ps)
**200 nm Ag**	-0.024 ± 0.008	6.7 ± 0.9
**4 p-CNS**	-0.093 ± 0.009	26.7 ± 4.6

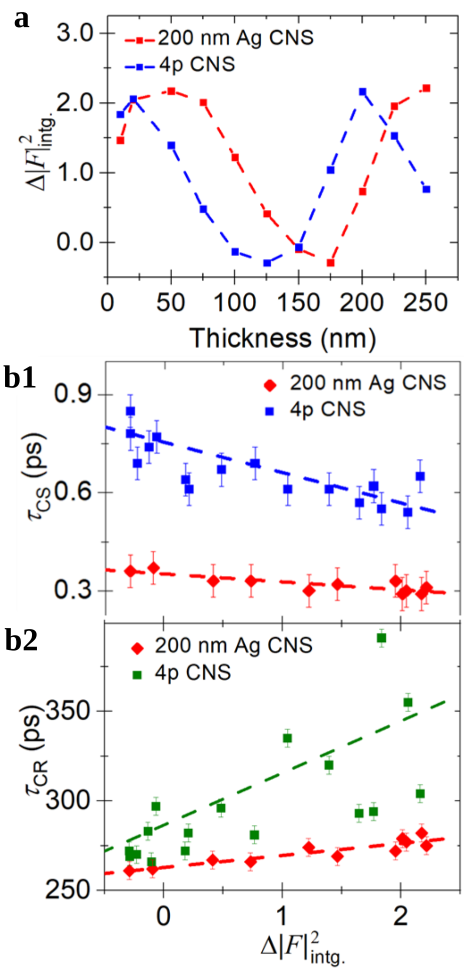

SI-Figure 20 | Nanophotonic control of CT dynamics induced by the optical field intensity. a, Modulations of the integral of the optical field intensity on 200 nm Ag thick film (■) and 4p Ag-Al_2O_3 CNS (■) substrates covered by Al_2O_3 top-covers of different thicknesses; the dashed lines are guides-for-the-eyes. **b,** CS (**b1**) and CR (**b2**) dynamics plotted against the integral of the optical field intensity. The dashed lines are guide-for-the-eyes. The optical field intensity was determined for a 725 nm incident beam and integrated across the D:A film. The measurements were completed in reflectance and transmittance modes for the Ag thick film and Ag-Al_2O_3 CNSs, respectively.

SI-VI.4. Tuning the Probe Beam Power and Diameter

To demonstrate further that the optical field analysis is the main parameter tuning charge transfer dynamics, control experiments were completed with probe beams of higher power and larger diameter. Because these parameters only allow a relatively moderate variation of *F*, when compared with engineering substrates, we focused on CR. SI-Figure 21 | and SI-Table 3 show that CR varies linearly with the optical field intensity.

SI-Table 3. Charge recombination (**τ_{CR}**) characteristic times obtained in TA transmittance mode on 4p Ag-Al_2O_3 CNSs covered with 125 nm thick Al_2O_3, and receiving different probe beam powers (*P*) and diameters (*D*).

D (mm)	*P* (mW)	τ_{CR} (ps)
0.2	0.2	269 ± 5 ^{T}
0.3	0.2	262 ± 5 ^{T}
0.4	0.2	252 ± 5 ^{T}
0.2	0.2	270 ± 5 ^{R}
0.2	0.1	263 ± 5 ^{R}
0.2	0.04	254 ± 5 ^{R}

Measured with 100 fs time interval in transmittance (^{T}) and reflectance (^{R}) modes.

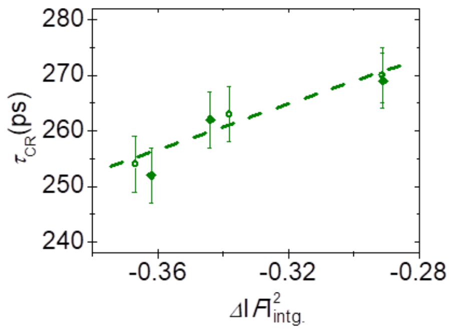

SI-Figure 21 | Nanophotonic effect on CR dynamics induced by tuning the probe beam characteristics. CR dynamics plotted against the integral of the optical field intensity for different probe beam powers (empty disks, o) and diameters (filled diamonds, ♦). The dashed line is a guide-for-the-eyes. The optical field intensity was calculated for a 725 nm incident beam and integrated across the D:A film. The measurements were completed in transmittance modes on top of Ag-Al_2O_3 CNSs covered with 125 nm thick Al_2O_3.

The obtained variation of τ_{CR} is larger than the experimental and fit uncertainties, while the modulations of the optical field intensity tuned with the power and the beam size lead to consistent charge transfer dynamics. The resulting slope is ~ 215 ± 84 ps, i.e. larger than the variation deduced from the Al_2O_3 thickness alteration stated in SI-Table 2.

SI-VI.5. Relative Contribution of the Number of Pairs on the Optical Field

We have also calculated the optical field intensity variation for 1, 2, 3 Ag-Al_2O_3 pairs with 10 nm thick Al_2O_3 top layer, which have been studied in a previous work.[18] The labelled red set of data point in SI-Figure 22 | shows that the number of pair increases both the IDI effect as well as $\Delta|F|^2$.

Noticeably, increasing the number of pair only increases $\Delta|F|^2$. Consequently, increasing the number of pairs once the increase of $\Delta|F|^2$ is taken into account corresponds to the red parabola in

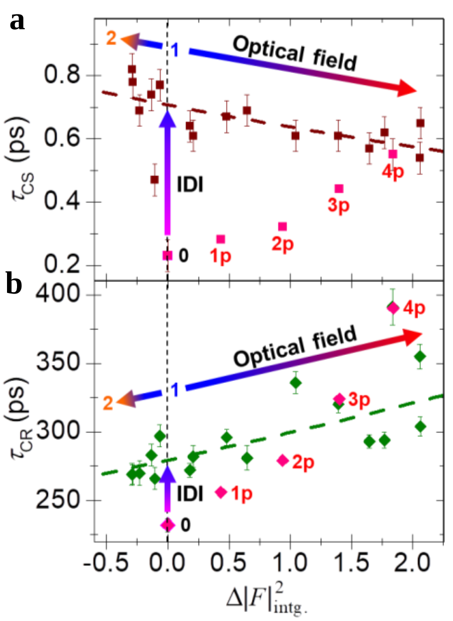

SI-Figure 22 | Relative effects of the number of pairs and optical field variations. a, CS and **b,** CR dynamics plotted against the integral of the optical field intensity. The brown and green data points were obtained by increasing the Ag-Al_2O_3 spacer thickness on-top of 4p CNSs, while the red data points were obtained by increasing the number of pair in 10 nm Al_2O_3 top cover CNSs. The dashed lines are guide-for-the-eyes. The optical field intensity was determined for a 725 nm incident beam and integrated across the D:A film.

SI-Figure 14 |b. The qualitative interpretation initially proposed remain valid as both CS and CR are slowed down when increasing the number of pairs.

SI-VI.6. Effect of the Donor:Acceptor Film Thickness

Finally, we note that when using CNSs to control charge transfer dynamics in D:A films, the thickness of the semiconductor materials is also important. SI-Figure 23 |a shows how varying the film thickness by about 20 nm impacts the modulation of the optical field intensity. Our experimental data are in good agreement with 60 nm thick D:A films as measured with a dektak profilometer. For thicker semiconductor films, the amplitude of the variations of $|F|^2$ is slightly decreased, whilst the period of its modulation is increased. From these calculations, the thickness of the D:A films is shown to be both a valid consistency check and an extra parameter allowing to further control charge transfer dynamics near CNSs. The thickness of the D:A film is then a tuning parameter to be added to the image dipole interactions and reflectance of the substrates.

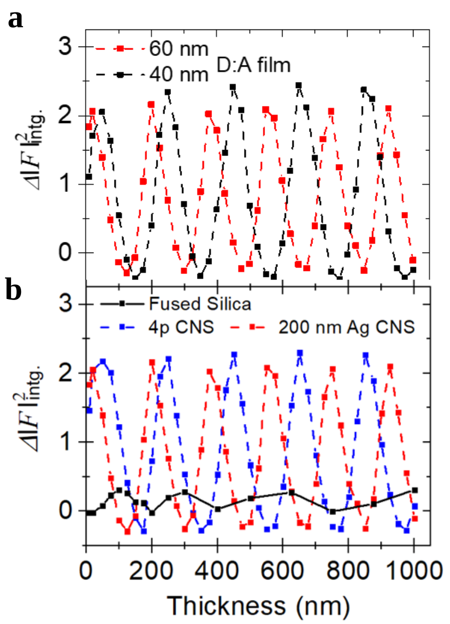

SI-Figure 23 | Nanophotonic control of the optical field intensity near selected substrates. a, Modulations of the integral of the optical field intensity, $|F|^2$, across 60 (■) and 40 (■) nm TriPh:PerDi thick films. **b,** comparison of $|F|^2$, modulation as a function of the Al_2O_3 cover thickness for fused silica (■), 4p Ag-Al_2O_3 CNS (■) and 200 nm Ag thick film (■) substrates. The dashed lines are guides-for-the-eyes. The optical field intensity was calculated for 4p Ag:Al_2O_3 CNSs, a 725 nm incident beam and integrated across the thickness of the D:A film.

SI-VII. Comments and Outlook

Considering the limit of the present model and data, we note that, CR data points present a wider dispersion from a linear variation with $\Delta|F|^2$ as compared to CS data point. This dispersion observed at large optical field intensity could result from sample fabrication and approximations of the model. For instance, *i*) a partial screening of the image dipoles could result from intrinsic doping in thick Al_2O_3 dielectric covers, or *ii*) defects in both metal and dielectric layers during the fabrication could result in scattering and alteration of the reflectivity coefficients, which would then alter the optical field intensity. In addition, we have plotted the CTD data as a function of the amplitude of the optical field to evidence a qualitative agreement between the model and the experiments. By doing so, it is implicit that the variation of the IDI effect with the separation between the dipoles and their images (SI-Figure 10 |) is neglected in terms like $\delta G_{\mathrm{CT}}^{\mathrm{IDI}}$ and $G_{\lambda-\mathrm{CT}}^{\mathrm{IDI}}$ included in k_{CT}^{IDI} and δ_{F2}, respectively. The impact of this effect can first be seen when focussing on the CNS data, where CS and CR present a very different dispersion of the data points. In the present system, CS is far from the barrierless point and presents a negligible deviation from a linear variation when its characteristic times are plotted as a function of $\Delta|F|^2_{\mathrm{intg}}$ (SI-Figure 13 |a), which supports the analysis model herein developed. In contrast, CR is close to the barrierless point, i.e. where $\Delta G_{CT} + \lambda_{CT} \approx 0$, and it presents a larger data point dispersion (SI-Figure 13 |b), with the thinnest spacers corresponding to the larger deviation from the linearity. In other words, the thinner spacers are associated with the largest IDI effects, which then deviate more strongly from the qualitative $\Delta|F|^2_{\mathrm{intg}}$ analysis. This CR behaviour is also consistent with the fact that close to the barrierless point any perturbation will have a relative strong impact when compared to $G_{\lambda-\mathrm{CT}}^{0} \cong 0$. Furthermore and adding to the consistency of this analysis, it can be seen when comparing SI-Figure 20 | and SI-Figure 13 | that single metal layer based CNSs, hence with a relatively weak IDI effect, present both a weaker NEOF effect but also less data point dispersion when compared with 4p CNSs. We also note that SI-Figure 21 | was obtained with the same with 125 nm thick Al_2O_3 based CNS, i.e. there is no variation of IDI amplitude in this figure. As the beam diameter and power are tuned, the resulting linear variation of the CTD as function of the optical field intensity gives further consistency to the argument developed above.

Finally, the model assumes that only Gibbs free energy and external reorganisation energy are affected by the optical field. However, we note that recent numerical works point at D:A molecular systems in which charge transfer integral can be affected by a static external electric field.[99-102] Whilst beyond the scope of the present manuscript, this is exciting from the point of view of experimental and numerical D:A material design opportunities.

The present work first reveals the NEOF effect, which to the best of our knowledge, had not been observed before, and next builds a theoretical analysis, which provides a qualitative agreement with the experiments. Reaching a quantitative description is beyond the goal of the present work, even though the above discussion points at specific directions that should be explored in the future.

Along similar lines of thought, we note that varying the temperature should, in first approximation, alter neither the IDI effect nor the optical field intensity. Consequently, combining temperature CTD measurements on NEOF substrates and appropriate fitting procedures could be a way to access Donor:Acceptor characteristics including intrinsic driving force and reorganisation energy in the solid state, i.e. where it is the most useful for the rational design of optoelectronic devices.

SI-VIII. Glossary

a : thickness of the metal and dielectric layer in the CNS, i.e. half of the CNS period.
b = *a* / *d* : normalised dipole length-CNS thickness layer.
CNS : composite nanostructure.
CR : charge recombination.
CS : charge separation.
CT : charge transfer.
CTD : charge-transfer dynamic.
d : dipole length of CT state inside D:A film.
D:A : donor:acceptor.
$f = F/F_{\mathrm{ext}}$: local field correction, internal to external electric field ratio.
f_v : metal volume fraction in the CNSs.
F : optical field.
$|F|^2$: optical field intensity, i.e. light intensity.
$F_{\mathrm{intg.}}$: optical field integrated over the thickness of a D:A thin film.

$|F|^2_{\text{intg.}}$: optical field intensity integrated across the D:A thin film.

$\Delta|F|^2_{\text{intg.}}$: difference of optical field intensity integrated across the D:A thin film between CNS and fused silica substrates.

$G^{\text{IDI}}_{\lambda-\text{CT}} = \Delta G^0_{\text{CT}} + \lambda^0_{\text{CT}} + \delta G^{\text{IDI}}_{\text{CT}}$: IDI altered intrinsic Gibbs free energy gain, IDI altered intrinsic driving force of the D:A system under consideration.

ΔG_{CT} : Gibbs free energy gain, driving force of a CT state.

ΔG^*_{CT} : activation Gibbs free energy of a CT state.

ΔG^0_{CT} : intrinsic Gibb free energy gain of a CT state, i.e. independent of both optical field and image dipole interactions resulting from the substrate.

$\delta G^{\text{IDI}}_{\text{CT}}$: Gibb free energy perturbation associated with a CT state dipoles and their image potentials within a substrate.

$\Delta G^{\text{NEOF}}_{\text{CT}}(p,F) = \Delta G^0_{\text{CT}} + \delta G^{\text{IDI}}_{\text{CT}}(p) - \Delta\vec{\mu}.\vec{F}$: Gibb free energy of a CT state affected by a nonlocal enhanced optical field effect.

h : distance from the top metal layer interface of the substrate across the dielectric top cover.

k_BT : thermal energy.

k_{CT} : charge-transfer-reaction rate.

IDI : image-dipole interactions.

ℓ : thickness of the alkyl melt separating the donor and acceptor columns from the Al_2O_3 top interface of the substrates as measured by grazing incidence wide angle X-ray scattering (GIXS).

L : thickness of the D:A film as measured by Dektak.

NEOF : nonlocal enhanced optical field.

P : power of an optical beam.

p : number of metal dielectric pairs in the CNSs.

PerDi : perylene diimide.

q_{DA} : the atomic charges of the donor and the acceptor.

$r = z / d$: normalised dipole-CNS interface distance.

$r_{\text{D+/A-}}$: ionic radii of the donor and the acceptor.

r_p and r_s: ellipsometry reflection coefficients for p- and s- polarisations, respectively.

R_{cc} : center-to-center distance between the donor and the acceptor, r_{DA} : the separation of the donor and the acceptor.

R : reflectance.

$\Delta R/R$: relative transmittance.

SPV : surface photovoltage.

T : transmittance.

$\Delta T/T$: relative transmittance.

TA : transient absorption.

$t_{\text{m,d}}$: thickness of the metal and dielectric in a CNS.

TriPh : triphenylene.

V_{DA} : electronic coupling between the initial and final states, charge-transfer integral.

z : distance from the top dielectric interface of the substrate to the dipole across the semiconductor donor:acceptor thin film.

$\Delta G^*_{\text{CT}}\Delta G^0_{\text{CT}}\delta G^{\text{IDI}}_{\text{CT}}\Delta G^{\text{NEOF}}_{\text{CT}}(p,F) = \Delta G^0_{\text{CT}} + \delta G^{\text{IDI}}_{\text{CT}}(p) - \Delta\vec{\mu}.\vec{F}\hat{\varepsilon}^{\text{IDI}}$: image-dipole interactions manipulated dielectric constant, including image-dipole interactions resulting from the dipole being located nearby the CNS.

ε_d : permittivity of the dielectric.

ε_m : permittivity of the metal.

ε_0 : permittivity of the vacuum.

$\varepsilon_{\text{optics}} = n^2$: optical-dielectric constant of the surrounding media.

$\varepsilon_{\text{static}}$: static dielectric constant of the surrounding media.

ϕ : diameter of an optical beam.

ϕ_γ : dimensionless energy parameter

$$\phi = \frac{U_{\text{tot}}}{U_0} = 1 + \frac{U^{\text{int.}}_\gamma}{U_0}$$

$$\phi^{\text{substrate}}_\gamma = \gamma\phi^{\text{substrate}}_{\text{paral.}} + (1-\gamma)\phi^{\text{substrate}}_{\text{perp.}}$$

Φ : dimensionless energy parameter integrated over the D:A film thickness.

$$\Phi^{\text{substrate}}_\gamma = \frac{1}{\text{L} - \lambda}\int_\lambda^{\text{L}} \phi^{\text{substrate}}_\gamma \text{dr}$$

$\Delta\Phi$: difference between CNS and Al_2O_3 dimensionless energy parameters integrated over the D:A film thickness.

$$\Delta\Phi^{\text{IDI}}_\gamma = \frac{1}{\text{L} - \lambda}\int_\lambda^{\text{L}} \left(\phi^{\text{IDI}}_\gamma - \phi^{\text{Al}_2\text{O}_3}_\gamma\right) \text{dr}$$

γ : dipole orientation with respect to metal interface with parallel ($\gamma = 1$), perpendicular ($\gamma = 0$), isotropic ($\gamma = 2/3$).

λ : wavelength of an incident optical beam to pump or probe the samples.

λ^0_{CT} : intrinsic reorganisation energy associated with charge transfer.

λ_{CT} : reorganisation energy associated with a charge transfer.

$\lambda^{\text{NEOF}}_{\text{CT}}(F) = \lambda^0_{\text{CT}} - \Delta\vec{\mu}.\vec{F}$: reorganisation energy associated with a charge transfer affected by a nonlocal enhanced optical field effect.

λ_{env} : external reorganisation energy accounting for reorientation and change of polarisation around the reagents upon CT.

$\lambda_{\text{intra.}}$: an intramolecular reorganisation energy accounting for vibrational, bond length and angles change within the reagents.

μ : dipole moment.

$\Delta\vec{\mu}$: difference between the dipole moment vectors in the initial and final charge transfer states.

θ : the angle between the CT dipole and the optical field.

$\tau_{\text{CT}} = 1/k_{\text{CT}}$: charge transfer reaction characteristic time as a function of the CT rate.

Ψ and Δ : measured ellipsometric angles defined from the ratio of the reflection coefficients r_p and r_s for the p- and s- polarisations, respectively.

SI-IX. Supplementary References

1. Coropceanu, V., *et al.*, Charge transport in organic semiconductors. *Chem. Rev.* **107**, 926-952 (2007).
2. Sokolov, A. N., *et al.*, From computational discovery to experimental characterization of a high hole mobility organic crystal. *Nat. Commun.* **2**, 437-445 (2011).
3. Ribierre, J. C., Aoyama, T., Muto, T., André, P., Hybrid Organic-Inorganic Liquid Bistable Memory Devices. *Org. Electron.* **12**, 1800-1805 (2011).
4. Dimitrov, S. D., Durrant, J. R., Materials Design Considerations for Charge Generation in Organic Solar Cells. *Chem. Mater.* **26**, 616-630 (2014).
5. Gorczak, N., *et al.*, Different mechanisms for hole and electron transfer along identical molecular bridges: the importance of the initial state delocalization. *J. Phys. Chem. A* **118**, 3891-3898 (2014).
6. Kim, R. H., *et al.*, Non-volatile organic memory with sub-millimetre bending radius. *Nat. Commun.* **5**, 3583-3595 (2014).
7. Muhieddine, K., Ullah, M., Pal, B. N., Burn, P., Namdas, E. B., All Solution‐Processed, Hybrid Light Emitting Field‐Effect Transistors. *Adv. Mater.* **26**, 6410-6415 (2014).
8. Howells, C. T., *et al.*, Enhanced organic solar cells efficiency through electronic and electro-optic effects resulting from charge transfers in polymer hole transport blends. *J. Mater. Chem. A* **4**, 4252-4263 (2016).
9. Ribierre, J. C., *et al.*, Ambipolar organic field-effect transistors based on solution-processed single crystal microwires of a quinoidal oligothiophene derivative. *Chem. Comm.* **51**, 5836-5839 (2015).
10. Howells, C. T., *et al.*, Influence of Perfluorinated Ionomer in PEDOT:PSS on the Rectification and Degradation of Organic Photovoltaic Cells. *J. Mater. Chem. A* **6**, 16012-16028 (2018).
11. Elemans, J. A. A. W., van Hameren, R., Nolte, R. J. M., Rowan, A. E., Molecular Materials by Self-Assembly of Porphyrins, Phthalocyanines, and Perylenes. *Adv. Mater.* **18**, 1251-1266 (2006).
12. Hayashi, H., *et al.*, Segregated donor-acceptor columns in liquid crystals that exhibit highly efficient ambipolar charge transport. *J. Am. Chem. Soc.* **133**, 10736-10739 (2011).
13. Schwartz, P. O., *et al.*, Perylenediimide-based donor-acceptor dyads and triads: impact of molecular architecture on self-assembling properties. *J. Am. Chem. Soc.* **136**, 5981-5992 (2014).
14. Squillaci, M. A., *et al.*, Self-Assembly of an Amphiphilic pi-Conjugated Dyad into Fibers: Ultrafast and Ultrasensitive Humidity Sensor. *Adv. Mater.* **27**, 3170-3174 (2015).

15. Zhao, K. Q., *et al.*, Highly Segregated Lamello-Columnar Mesophase Organizations and Fast Charge Carrier Mobility in New Discotic Donor-Acceptor Triads. *Chem. Eur. J. A* **21**, 10379-10390 (2015).
16. Sun, K., *et al.*, A molecular nematic liquid crystalline material for high-performance organic photovoltaics. *Nat. Commun.* **6**, 6013-6022 (2015).
17. Sergeyev, S., Pisula, W., Geerts, Y. H., Discotic liquid crystals: a new generation of organic semiconductors. *Chem. Soc. Rev.* **36**, 1902-1929 (2007).
18. Lee, K. J., *et al.*, Charge-transfer dynamics and nonlocal dielectric permittivity tuned with metamaterial structures as solvent analogues. *Nat. Mater.* **16**, 722-729 (2017).
19. Xiao, Y., *et al.*, Chemical engineering of donor-acceptor liquid crystalline dyads and triads for the controlled nanostructuration of organic semiconductors. *Crystengcomm* **18**, 4787-4798 (2016).
20. Lee, K. J., *et al.*, Structure–charge transfer property relationship in self-assembled discotic liquid-crystalline donor–acceptor dyad and triad thin films. *RSC Adv.* **6**, 57811-57819 (2016).
21. Chen, L. X., Xiao, S. Q., Yu, L. P., Dynamics of photoinduced electron transfer in a molecular donor-acceptor quartet. *J. Phys. Chem. B* **110**, 11730-11738 (2006).
22. Roland, T., *et al.*, Sub-100 fs charge transfer in a novel donor-acceptor-donor triad organized in a smectic film. *Phys. Chem. Chem. Phys.* **14**, 273-279 (2012).
23. Sartin, M. M., *et al.*, Nonlinear Optical Pulse Suppression via Ultrafast Photoinduced Electron Transfer in an Aggregated Perylene Diimide/Oligothiophene Molecular Triad. *J. Phys. Chem. A* **118**, 110-121 (2014).
24. Beyreuther, E., Grafstrom, S., Eng, L. M., Designing a Robust Kelvin Probe Setup Optimized for Long-Term Surface Photovoltage Acquisition. *Sensors* **18**, 4068 (2018).
25. Beyreuther, E., Becherer, J., Thiessen, A., Grafström, S., Eng, L. M., Electronic surface properties of SrTiO3 derived from a surface photovoltage study. *Surf. Science* **612**, 1-9 (2013).
26. Robertson, J., Falabretti, B., Band offsets of high K gate oxides on III-V semiconductors. *J. Appl. Phys.* **100**, 014111 (2006).
27. Eremeev, S. V., Schmauder, S., Hocker, S., Kulkova, S. E., Investigation of the electronic structure of Me/Al2O3(0001) interfaces. *Physica B* **404**, 2065-2071 (2009).
28. Zhukovskii, Y. F., Kotomin, E. A., Herschend, B., Hermansson, K., Jacobs, P. W. M., The adhesion properties of the Ag/α-Al2O3 interface: an ab initio study. *Surf. Science* **513**, 343-358 (2002).
29. Deng, H., *et al.*, Nature of Ag Species on Ag/γ-Al2O3: A Combined Experimental and Theoretical Study. *ACS Catal.* **4**, 2776−2784 (2014).
30. Jellison, G. E., Modine, F. A., Parameterization of the optical functions of amorphous materials in the interband region. *Appl. Phys. Lett.* **69**, 371-374 (1996).
31. Kim, K., Lim, H., Lee, D.-H., Invariant Imbedding Equations for Electromagnetic Waves in Stratified Magnetic Media: Applications to One-Dimensional Photonic Crystals. *J. Korean Phys. Soc.* **39**, L956-L960 (2001).
32. Lee, K. J., Wu, J. W., Kim, K., Enhanced nonlinear optical effects due to the excitation of optical Tamm plasmon polaritons in one-dimensional photonic crystal structures. *Opt. Express* **21**, 28817-28823 (2013).
33. Lee, K. J., Lee, Y. U., Kim, S. J., André, P., Hyperbolic Dispersion Dominant Regime Identified through Spontaneous Emission Variations near Metamaterial Interfaces. *Adv. Mater. Interfaces* **5**, 1701629 (2018).
34. Paudel, H. P., Bayat, K., Baroughi, M. F., May, S., Galipeau, D. W., Geometry dependence of field enhancement in 2D metallic photonic crystals. **17**, 22179-22189 (2009).
35. Devizis, A., *et al.*, Dissociation of Charge Transfer States and Carrier Separation in Bilayer Organic Solar Cells: A Time-Resolved Electroabsorption Spectroscopy Study. *J. Am. Chem. Soc.* **137**, 8192-8198 (2015).
36. Wilcox, D. E., *et al.*, Ultrafast Charge-Transfer Dynamics at the Boron Subphthalocyanine Chloride/C60 Heterojunction: Comparison between Experiment and Theory. *J. Phys. Chem. Lett.* **6**, 569-575 (2015).
37. Su, C. Y., *et al.*, Dependencies of surface plasmon coupling effects on the p-GaN thickness of a thin-p-type light-emitting diode. **25**, 21526-21536 (2017).
38. Kavarnos, G. J., Turro, N. J., Photosensitization by Reversible Electron Transfer: Theories, Experimental Evidence, and Examples. *Chem. Rev.* **86**, 401-449 (1986).
39. Oevering, H., *et al.*, Long-range photoinduced through-bond electron transfer and radiative recombination via rigid nonconjugated bridges: distance and solvent dependence. *J. Am. Chem. Soc.* **109**, 3258-3269 (1987).
40. Rosspeintner, A., Vauthey, E., Bimolecular photoinduced electron transfer reactions in liquids under the gaze of ultrafast spectroscopy. *Phys. Chem. Chem. Phys.* **16**, 25741-25754 (2014).
41. Kircher, T., Löhmannsröben, H. G., Photoinduced charge recombination reactions of a perylene dye in acetonitrile. *Phys. Chem. Chem. Phys.* **1**, 3987-3992 (1999).
42. Holman, M. W., *et al.*, Studying and switching electron transfer: from the ensemble to the single molecule. *J. Am. Chem. Soc.* **126**, 16126-16133 (2004).
43. Hofmann, C. C., Bauer, P., Haque, S. A., Thelakkat, M., Kohler, J., Energy- and charge-transfer processes in flexible organic donor-acceptor dyads. *J. Chem. Phys.* **131**, 144512 (2009).
44. Bagui, M., *et al.*, Synthesis and optical properties of triphenylene-based dendritic donor perylene diimide acceptor systems. *J. Phys. Chem. A* **115**, 1579-1592 (2011).
45. Lemaur, V., *et al.*, Charge transport properties in discotic liquid crystals: a quantum-chemical insight into structure-property relationships. *J. Am. Chem. Soc.* **126**, 3271-3279 (2004).
46. Lemaur, V., Steel, M., Beljonne, D., Bredas, J. L., Cornil, J., Photoinduced charge generation and recombination dynamics in model donor/acceptor pairs for organic solar cell applications: A full quantum-chemical treatment. *J. Am. Chem. Soc.* **127**, 6077-6086 (2005).
47. Beljonne, D., *et al.*, Electronic Processes at Organic−Organic Interfaces: Insight from Modeling and Implications for Opto-electronic Devices. *Chem. Mater.* **23**, 591-609 (2011).
48. Datta, A., Mohakud, S., Pati, S. K., Comparing the electron and hole mobilities in the α and β phases of perylene: role of π-stacking. *J. Mater. Chem.* **17**, 1933-1938 (2007).
49. Bromley, S. T., Illas, F., Mas-Torrent, M., Dependence of charge transfer reorganization energy on carrier localisation in organic molecular crystals. *Phys. Chem. Chem. Phys.* **10**, 121-127 (2008).
50. Chang, T. L., Cheung, H. C., Solvent effects on the photoisomerization rates of the zwitterionic and the cationic forms of rhodamine B in protic solvents. *J. Phys. Chem.* **96**, 4874–4878 (1992).
51. André, P., *et al.*, Hybrid Dendritic Molecules with Confined Chromophore Architecture to Tune Fluorescence Efficiency. *J. Phys. Chem. B* **112**, 16382-16392 (2008).
52. Vautravers, N. R., André, P., Cole-Hamilton, D. J., Fluorescence Activation of a Polyhedral Oligomeric Silsesquioxane in the Presence of Reducing Agents. *J. Mater. Chem* **19**, 4545-4550 (2009).
53. Vautravers, N. R., Andre, P., Slawin, A. M. Z., Cole-Hamilton, D. J., Synthesis and characterization of photoluminescent vinylbiphenyl decorated polyhedral oligomeric silsesquioxanes. *Org. Biomol. Chem.* **7**, 717-724 (2009).
54. Chen, S., Hoskins, C., Wang, L., MacDonald, M. P., André, P., A Water-Soluble Temperature nanoProbe based on a Multimodal Magnetic-Luminescent nanoColloid *Chem. Comm.* **48**, 2501-2503 (2012).
55. Van der Auweraer, M., Van den Zegel, M., Boens, N., Deschryver, F. C., Willig, F., Photophysics of 2-Phenyl-3-Indolocarbocyanine Dyes. *J. Phys. Chem.* **90**, 1169-1175 (1986).
56. Sauerwein, B., Murphy, S., Schuster, G. B., Dynamics of Solute Motion - Photoisomerization Shows Linear-Dependence on Solvent Mass. *J. Am. Chem. Soc.* **114**, 7920-7922 (1992).
57. Murphy, S., Schuster, G. B., Electronic Relaxation in a Series of Cyanine Dyes - Evidence for Electronic and Steric Control of the Rotational Rate. *J. Phys. Chem.* **99**, 8516-8518 (1995).
58. Drexhage, K. H., Kuhn, H., Schäfer, F. P., Variation of the Fluorescence Decay Time of a Molecule in Front of a Mirror. *Ber. Bunsenges. Phys.* **72**, 329 (1968).
59. Morawitz, H., Self-Coupling of a Two-Level System by a Mirror. *Phys. Rev.* **187**, 1792-1796 (1969).
60. Drexhage, K. H., Influence of a dielectric interface on fluorescence decay time. *J. Lumin.* **1-2**, 693-701 (1970).
61. Kuhn, H., Classical Aspects of Energy Transfer in Molecular Systems. *J. Chem. Phys.* **53**, 101 (1970).
62. Chance, R. R., Prock, A., Silbey, R., Lifetime of an excited molecule near a metal mirror: Energy transfer in the Eu3+ / silver system. *J. Chem. Phys.* **60**, 2184 (1974).

63. Chance, R. R., Miller, A. H., Prock, A., Silbey, R., Luminescent Lifetimes Near Multiple Interfaces: A Quantitative Comparison of Theory and Experiment. *Chem. Phys. Lett.* **33**, 590-592 (1975).
64. Chance, R. R., Prock, A., Silbey, R., in *Advances in Chemical Physics*, Prigogine, I., Rice, S.A. Eds. (1978), vol. 37, pp. 1-65.
65. Amos, R. M., Barnes, W. L., Modification of the spontaneous emission rate of Eu3+ ions close to a thin metal mirror. *Phys. Rev. B* **55**, 7249-7254 (1997).
66. Barnes, W. L., Fluorescence near interfaces: The role of photonic mode density. *J. Mod. Opt.* **45**, 661-699 (1998).
67. Worthing, P. T., Amos, R. M., Barnes, W. L., Modification of the spontaneous emission rate of Eu3+ ions embedded within a dielectric layer above a silver mirror. *Phys. Rev. A* **59**, 865-872 (1999).
68. Amos, R. M., Barnes, W. L., Modification of spontaneous emission lifetimes in the presence of corrugated metallic surfaces. *Phys. Rev. B* **59**, 7708-7714 (1999).
69. Astilean, S., Barnes, W. L., Quantum efficiency and the photonic control of molecular fluorescence in the solid state. *Appl. Phys. B* **75**, 591-594 (2002).
70. Astilean, S., Garrett, S., Andrew, P., Barnes, W. L., Controlling the fluorescence lifetime of dyes in nanostructured geometries. *J. Mol. Struct.* **651**, 277-283 (2003).
71. Blum, C., *et al.*, Nanophotonic Control of the Förster Resonance Energy Transfer Efficiency. *Phys. Rev. Lett.* **109**, 203601-203605 (2012).
72. Bovensiepen, U., Petek, H., Wolf, M., *Dynamics at Solid State Surfaces and Interfaces* (Wiley, 2012), pp. 237.
73. Franzen, S., Boxer, S. G., in *Electron Transfer in Inorganic, Organic, and Biological Systems*, Bolton, J.R., Mataga, N., McLendon, G. Eds. (American Chemical Society, 1991), vol. 228, pp. 149-162.
74. Tanaka, S., Marcus, R. A., Electron Transfer Model for the Electric Field Effect on Quantum Yield of Charge Separation in Bacterial Photosynthetic Reaction Centers. *J. Phys. Chem. B* **101**, 5031-5045 (1997).
75. Murgida, D. H., Hildebrandt, P., Electrostatic-Field Dependent Activation Energies Modulate Electron Transfer of Cytochrome c. *J. Phys. Chem. B* **106**, 12814-12819 (2002).
76. Kranich, A., Ly, H. K., Hildebrandt, P., Murgida, D. H., Direct Observation of the Gating Step in Protein Electron Transfer: Electric-Field-Controlled Protein Dynamics. *J. Am. Chem. Soc.* **130**, 9844-9848 (2008).
77. Ohta, N., Electric-field effects on photoinduced dynamics and function. *Pure Appl. Chem.* **85**, 1427-1435 (2013).
78. Boxer, S. G., Stark Realities. *J. Phys. Chem. B* **113**, 2972-2983 (2009).
79. Ohta, N., *et al.*, Acceleration and deceleration of photoinduced electron transfer rates by an electric field in porphyrin-fullerence dyads. *Chem. Phys. Lett.* **368**, 230-235 (2003).
80. Hilczer, M., Traytak, S., Tachiya, M., Electric field effects on fluorescence quenching due to electron transfer. *J. Chem. Phys.* **115**, 11249 (2001).
81. Hilczer, M., Tachiya, M., Electric field effects on fluorescence quenching due to electron transfer. II. Linked donor–acceptor systems. *J. Chem. Phys.* **117**, 1759 (2002).
82. Hilczer, M., Bandyopadhyay, T., Tachiya, M., Electric field effect on electron transfer between donor and acceptor in polymer matrix. *J. Photochem. Photobiol. A* **166**, 33-44 (2004).
83. Nakabayashi, T., Morikawa, T., Ohta, N., Direct measurements of the electric-field-induced change in fluorescence decay profile of pyrene doped in a polymer film. *Chem. Phys. Lett.* **395**, 346-350 (2004).
84. Lu, S.-Z., Li, X.-Y., Liu, J.-F., Effect of external electric field on electron transfer in conjugated molecular wire. *Chem. Phys.* **297**, 31-37 (2004).
85. Tsushima, M., Ohta, N., Electric field effects on photoinduced electron transfer processes of methylene-linked compounds of pyrene and N,N-dimethylaniline in a polymer film. *J. Chem. Phys.* **120**, 6238-6245 (2004).
86. Iimori, T., Yoshizawa, T., Nakabayashi, T., Ohta, N., Time-resolved measurements of the external electric field effects on fluorescence in electron donor and acceptor pairs of N-ethylcarbazole and dimethyl terephthalate doped in a polymer film. *Chem. Phys.* **319**, 101-110 (2005).
87. Ohara, Y., *et al.*, Electric-Field-Induced Changes in Absorption and Emission Spectra of CdS Nanoparticles Doped in a Polymer Film. *J. Phys. Chem. B* **110**, 20927-20936 (2006).
88. Yoshizawa, T., Mizoguchi, M., Iimori, T., Nakabayashi, T., Ohta, N., Effects of electric and magnetic fields on fluorescence in electron donor and acceptor pairs of pyrene and N-methylphthalimide doped in a polymer film. *Chem. Phys.* **324**, 26-39 (2006).
89. Zhou, H., *et al.*, Electric field induced fluorescence hysteresis of single molecules in poly(methyl methacrylate). *Appl. Phys. Lett.* **105**, 153301 (2014).
90. Mehata, M. S., Enhancement of Charge Transfer and Quenching of Photoluminescence of Capped CdS Quantum Dots. *Sci. Rep.* **5**, 12056-12067 (2015).
91. Ito, T., Yamazaki, I., Ohta, N., Distance Dependence of the Electric Field Effect on Photoinduced Electron Tunneling between Cyanine Dye and Viologen through a Fatty Acid Monolayer. *J. Phys. Chem. B* **106**, 895-898 (2002).
92. Ohta, N., Koizumi, M., Umeuchi, S., Nishimura, Y., Yamazaki, I., External Electric Field Effects on Fluorescence in an Electron Donor and Acceptor System: Ethylcarbazole and Dimethyl Terephthalate in PMMA Polymer Films. *J. Phys. Chem.* **100**, 16466-16471 (1996).
93. Ohta, N., Electric Field Effects on Photochemical Dynamics in Solid Films. *Bull. Chem. Soc. Jpn* **75**, 1637-1655 (2002).
94. Farazdel, A., Dupuis, M., Clementi, E., Aviram, A., Electric-field induced intramolecular electron transfer in spiro .pi.-electron systems and their suitability as molecular electronic devices. A theoretical study. *J. Am. Chem. Soc.* **112**, 4206-4214 (1990).
95. Seki, K., Tachiya, M., Electric field dependence of charge mobility in energetically disordered materials: Polaron aspects. *Phys. Rev. B* **65**, 014305 (2001).
96. Sancho-García, J. C., Horowitz, G., Brédas, J. L., Cornil, J., Effect of an external electric field on the charge transport parameters in organic molecular semiconductors. *J. Chem. Phys.* **119**, 12563 (2003).
97. Dhoot, A. S., Hogan, J. A., Morteani, A. C., Greenham, N. C., Electromodulation of photoinduced charge transfer in polyfluorene bilayer devices. *Appl. Phys. Lett.* **85**, 2256 (2004).
98. Ouyang, J. Y., Chu, C. W., Sieves, D., Yang, Y., Electric-field-induced charge transfer between gold nanoparticle and capping 2-naphthalenethiol and organic memory cells. *Appl. Phys. Lett.* **86**, 123507 (2005).
99. Song, P., Li, Y., Ma, F., Pullerits, T., Sun, M., External Electric Field-Dependent Photoinduced Charge Transfer in a Donor–Acceptor System for an Organic Solar Cell. *J. Phys. Chem. C* **117**, 15879-15889 (2013).
100. Li, Y., Feng, Y., Sun, M., Photoinduced Charge Transport in a BHJ Solar Cell Controlled by an External Electric Field. *Sci. Rep.* **5**, 13970 (2015).
101. Song, P., Li, Y., Ma, F., Sun, M., Insight into external electric field dependent photoinduced intermolecular charge transport in BHJ solar cell materials. *J. Mater. Chem. C* **3**, 4810-4819 (2015).
102. Song, P., Li, Y., Ma, F., Pullerits, T., Sun, M., Photoinduced Electron Transfer in Organic Solar Cells. *Chem. Rec.* **16**, 734-753 (2016).
103. Cappel, U. B., Feldt, S. M., Schoneboom, J., Hagfeldt, A., Boschloo, G., The influence of local electric fields on photoinduced absorption in dye-sensitized solar cells. *J. Am. Chem. Soc.* **132**, 9096-9101 (2010).
104. Oliveira, S. L., *et al.*, in *Quantum Electronics and Laser Science Conference (QELS)* Ed^'Eds. (OSA: Baltimore, Maryland, United States, 2005), vol. 2, pp. 1277-1279.
105. Correa, D. S., *et al.*, Investigation of the two-photon absorption cross-section in perylene tetracarboxylic derivatives: nonlinear spectra and molecular structure. *J. Phys. Chem. A* **110**, 6433-6438 (2006).
106. Huang, C., *et al.*, Photo-induced charge transfer and nonlinear absorption in dyads composed of a two-photon-absorbing donor and a perylene diimide acceptor. *J. Mater. Chem.* **21**, 16119-16128 (2011).
107. Huang, C., *et al.*, Photoinduced Electron Transfer and Nonlinear Absorption in Poly(carbazole-alt-2,7-fluorene)s Bearing Perylene Diimides as Pendant Acceptors. *J. Phys. Chem. A* **116**, 4305-4317 (2012).
